# Systematic integrated analyses of methylomic and transcriptomic impacts of early combined botanicals on estrogen receptor-negative mammary cancer

**DOI:** 10.1038/s41598-021-89131-5

**Published:** 2021-05-04

**Authors:** Itika Arora, Yuanyuan Li, Manvi Sharma, Michael R. Crowley, David K. Crossman, Shizhao Li, Trygve O. Tollefsbol

**Affiliations:** 1 Department of Biology, University of Alabama at Birmingham, 1300 University Boulevard, Birmingham, AL 35294 USA; 2 Department of Obstetrics and Gynecology and Women’s Health, University of Missouri, Columbia, MO 65201 USA; 3 Department of Surgery, University of Missouri, Columbia, MO 65212 USA; 4 Department of Genetics, University of Alabama at Birmingham, Birmingham, AL 35294 USA; 5 Comprehensive Center for Healthy Aging, University of Alabama Birmingham, 1530 3rd Avenue South, Birmingham, AL 35294 USA; 6 Comprehensive Cancer Institute, University of Alabama Birmingham, 1802 6th Avenue South, Birmingham, AL 35294 USA

**Keywords:** Cancer, Computational biology and bioinformatics

## Abstract

Dietary botanicals such as the cruciferous vegetable broccoli sprouts (BSp) as well as green tea polyphenols (GTPs) have shown exciting potential in preventing or delaying breast cancer (BC). However, little is known about their impact on epigenomic aberrations that are centrally involved in the initiation and progression of estrogen receptor-negative [ER(−)] BC. We have investigated the efficacy of combined BSp and GTPs diets on mammary tumor inhibition in transgenic Her2/neu mice that were administered the diets from prepubescence until adulthood. Herein, we present an integrated DNA methylome and transcriptome analyses for defining the early-life epigenetic impacts of combined BSp and GTPs on mammary tumors and our results indicate that a combinatorial administration of BSp and GTPs have a stronger impact at both transcriptome and methylome levels in comparison to BSp or GTPs administered alone. We also demonstrated a streamlined approach by performing an extensive preprocessing, quality assessment and downstream analyses on the genomic dataset. Our identification of differentially methylated regions in response to dietary botanicals administered during early-life will allow us to identify key genes and facilitate implementation of the subsequent downstream functional analyses on a genomic scale and various epigenetic modifications that are crucial in preventing ER(−) mammary cancer. Furthermore, our realtime PCR results were also found to be consistent with our genome-wide analysis results. These results could be exploited as a comprehensive resource for understanding understudied genes and their associated epigenetic modifications in response to these dietary botanicals.

## Introduction

Cancer is one of the leading causes of death in the United States. Based on the American Cancer Society statistics, in 2020, there were approximately 1,806,590 new cancer cases and 605,520 breast cancer (BC) cases. BC is highly prevalent and accounts for high mortality rates^1,2^. Steroid receptor hormones play a crucial role in the growth and development of the mammary glands in mice as well as in BC in humans. BC with active estrogen receptor (ER) expression is known as ER-positive BC [ER(+) BC], which is often treated by endocrine therapy. As a result, endocrine therapy reduces estrogen levels or blocks the estrogen-ER signaling pathway. Studies have shown that women with ER(+) BC normally have a good prognosis with lower recurrence rates^3^. Unlike ER(+) BC, ER(−) BC such as triple-negative BC (TNBC) does not express the ER, progesterone receptors and human epidermal growth factor 2 (*HER2*) gene. The treatment of TNBC is more challenging than ER(+) BC and the tumor progresses at a faster rate in comparison to ER(+) BC^4^. The absence of the ER gene renders it even more challenging to control, thereby confining the treatment options.

Consumption of dietary botanicals such as green tea and cruciferous vegetables demonstrates promising results in cancer prevention. Epidemiological studies have shown that higher consumption of various fruits and vegetables in countries like Asia, leads to a decline in BC incidence^5^. Another study in Mediterranean and Greek populations has provided strong evidence supporting the hypothesis that a diet high in fiber may reduce the risk of ER(−) BC^6^. This dietary pattern contains multiple protective substances such as high amounts of fiber, essential fatty acids and vitamins E and C, which may be associated with lowering the risk of BC^7^. Mechanistically, dietary polyphenols exhibit an active involvement in various cancer pathways such as the RTK/RAS, PI3K, p53 and cell signaling pathways, which may contribute to their chemopreventive effects on cancers^8^. Amongst various dietary botanicals, isothiocyanates in cruciferous vegetables such as cabbage, kale, cauliflower and broccoli sprouts (BSp) as well as green tea polyphenols (GTPs) play an active role in preventing cancer^9^. Our previous studies have suggested that the combined administration of epigallocatechin-3-gallate in GTPs and the isothiocyanate, sulforaphane in BSp led to synergistic inhibition of cellular proliferation^10^. Furthermore, this combinatorial dietary treatment also resulted in inhibition of tumor development in a BC xenograft mice model^10^. However, even with the recent advancement towards analyzing various anti-cancerous properties of these dietary phytochemicals, little is known about their impact on the epigenomic machinery in ER(−) BC.

Herein, we present an integrated analyses of reduced-representation bisulfite sequencing (RRBS) methylome analyses coupled with RNA-seq transcriptome analyses to evaluate whether early life consumption of combined BSp and GTPs is highly effective in neutralizing epigenomic aberrations and epigenetic control of key genes which can lead to inhibition of ER(−) BC formation and progression. Using a Her2/neu transgenic mammary cancer ER(−) mouse model, our results not only exhibited the combinatorial impacts of BSp and GTPs-induced global DNA methylomic changes, but also illuminated the impacts of genomic regulatory RNA changes on gene expression at specific life stages. Our DNA methylome analyses based on p value ≤ 0.05, identified 2874 (2252 hypermethylated vs. 622 hypomethylated) differentially methylated regions (DMGs) in tumors from mice treated with BSp, 4074 (3538 hypermethylated vs. 536 hypomethylated) DMGs from GTPs treatment and 4181 (3639 hypermethylated vs. 542 hypomethylated) DMGs in mice that received the combination (BSp + GTPs) treatment. Similarly, transcriptomic analyses based on p value ≤ 0.05, identified a total of 146 differentially expressed genes (DEGs) in the tumors from mice receiving BSp, no DEGs in the tumors from mice receiving GTPs and 895 DEGs in the tumors from mice receiving the combination treatment group.

Subsequently, 8 DMGs-DEGs correlated pairs were identified in the mammary tumors of mice receiving BSp treatment and 39 DMGs-DEGs correlated pairs were identified in the tumors of mice receiving the combinatorial (BSp + GTPs) diet. Our pathway analyses further revealed association of DEGs and DMGs with various epigenetics modifications such as DNA methylation and histone modifications (such as Histone H3K4 methylation, Histone H3-K4 trimethylation and histone acetylation). These results indicate that a combinatorial administration of BSp and GTPs has a stronger impact at both the transcriptome and methylome levels in comparison to BSp or GTPs alone. Thus, our dataset including both DNA methylomic and RNA-seq transcriptome analyses is very exclusive and could be of importance to future investigation by revealing the impact of these dietary combinations as well as other novel dietary combinations.

## Results

### The combinatorial treatment demonstrated higher efficacy in preventing ER(−) mammary tumor development in comparison to BSp and GTPs administered singly

**Figure 1:**
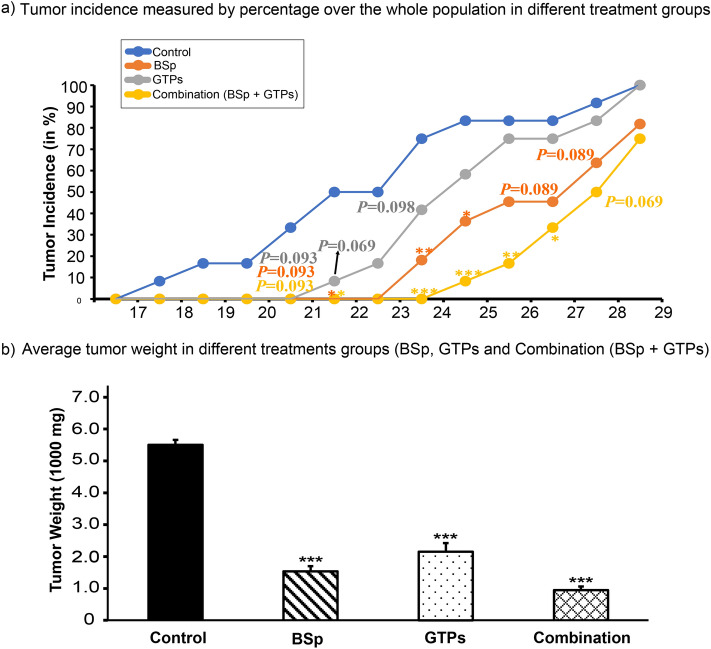
Her2/neu mice were administered with regular control diet, 26% BSp diet, 0.5% GTPs in drinking water or BSp and GTP in combination (BSp + GTPs) upon weaning at 3 weeks. Dietary treatment continued throughout the study until termination of the experiment and mice were evaluated for tumor growth weekly. (**a**) Tumor incidence measured by percentage over the whole population. (**b**) Average tumor weight among BSp, GTPs and the combination treatment group. Columns represents mean; bars, standard error; *p value < 0.05, **p value < 0.01, ***p value < 0.001 which were significantly different from the control group.

To observe tumor development in response to BSp, GTPs and combination (BSp + GTPs) treatments, we evaluated the effects of these treatment approaches in Her2/neu transgenic mouse model that develops spontaneous ER(−) mammary tumors driven by overexpression of an oncogene. We found that the combinatorial treatment group exhibited the strongest inhibitory effect on tumor growth (Fig. 1). The BSp and the combination treatment groups displayed a significant decline in tumor incidence from 21 weeks of age and thereafter (p value < 0.05) (Fig. 1a). Further, the weight of tumors was significantly decreased in the BSp, GTPs and combination (BSp + GTPs) treatment groups with p value < 0.001 (Fig. 1b). Early life BSp and/or GTPs administration had no effect on mouse body weight, food and water consumption of Her2/neu mice at the same point in the time (Supplementary Fig. S1). Subsequently, the BSp treatment group was more efficacious than the GTPs treatment group. Overall, the combination treatment group was most effective in suppressing the tumor development, as demonstrated by its lowest tumor incidence and smallest tumor weight in comparison to the BSp and GTPs treatment groups.

### Differential gene expression profile analyses induced by combined administration of BSp and GTPs

**Figure 2:**
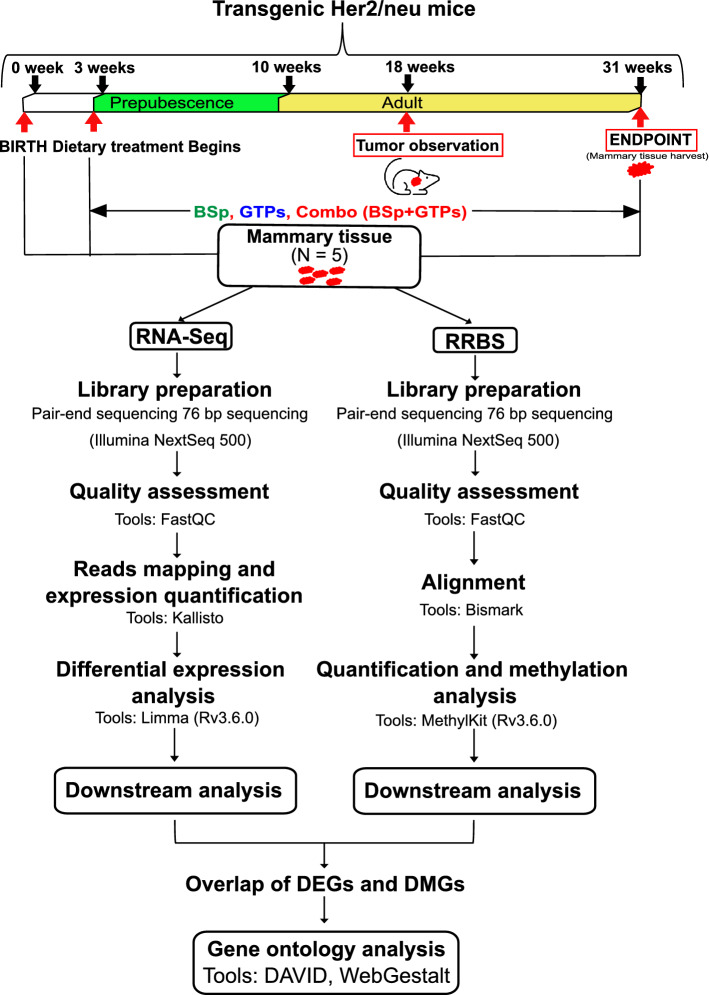
An overview of framework demonstrating experimental design and data analyses pipeline.

**Figure 3:**
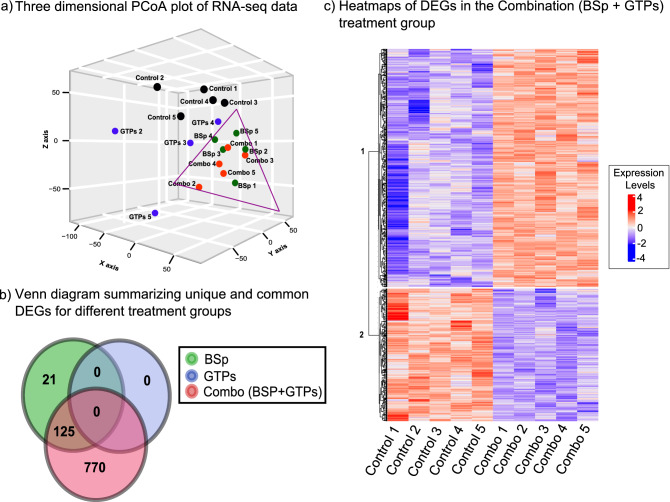
Transcriptome analyses across BSp, GTPs and Combination (BSp + GTPs) treatment groups. (**a**) Three dimensional PCoA plot demonstrating spatial arrangements of different samples. (**b**) Venn diagram summarizing total number of unique and overlapping differentially expressed genes in BSp, GTPs and combination (BSp + GTPs) treatment groups. Green circle represents BSp, Blue color represents GTPs and Red color denotes the combination (BSp + GTPs) treatment group. (**c**) Heatmap representing 895 up-regulated and down-regulated genes in the combination (BSp + GTPs) treatment group based on q value. Each row corresponds to differentially expressed transcripts and each column represents biological replicates in control (N_Control_ = 5) and the combination (N_Combination_ = 5) treatment group. Blue color denotes lower expression levels and red color denotes higher expression levels.

Our current studies and previous publications indicate that combinatorial treatment with BSp and GTPs exhibited the most significant preventive and inhibitory effects on BC in vitro compared to these two compounds that were administered singly. In order to better understand the global influence of combination dietary treatment compared to the individually administered BSp or GTPs, we evaluated the RNA expression using RNA sequencing (RNA-seq) analyses in mammary tumors of Her2/neu mice in the different treatment groups (Control-BSp, Control-GTPs, Control-Combination) as done previously^11^. The mammary tumor tissues were harvested at 31 weeks (endpoint) (N_Control_ = 5, N_BSp_ = 5, N_GTPs_ = 5, N_Combination_ = 5) (Fig. 2) and further sent for RNA-seq pair-end library preparation. Initially, the RNA-seq data were transformed for linear modeling and samples outliers were identified by generating a boxplot across all the samples in different treatment groups (Supplementary Fig. S2a). As a result, GTP1 displayed abnormal distribution and was removed from further processing/analyses (Supplementary Fig. S2b). The distribution of remaining samples was assessed by generating a histogram (Supplementary Fig. S2c). Furthermore, unsupervised principal component analyses (PCoA) were conducted on gene expression profiles for individual samples among different treatment groups to reveal gene expression shift (Fig. 3a). Based on the spatial arrangements in a PCoA plot, GTPs had no significant overlap; however, we noticed some overlaps among BSp and the combination treatment groups (Fig. 3a,b). The latter result was verified by differential expression analyses using qRT-PCR. Based on the spatial arrangements of GTPs, as expected, there were no transcripts which were differentially expressed (DE). However, gene expression was slightly changed among the BSp treatment group compared to the control group.

Among the total 14,157 transcripts (genes) detected, 146 (1.03%) genes were DE in BSp treatment with 90 (61.64%) genes up-regulated and 55 (38.35%) downregulated using a 5% false discovery rate (FDR) and fold-change (log_10_ FC) cutoff. In comparison to BSp and GTPs treatment alone, gene expression profiling was drastically changed among the combination treatment group. Out of 14,157 genes in the combination treatment, 895 (6.32%) genes were DE amongst which 575 (64.25%) genes were up-regulated and 320 (35.75%) genes were down-regulated using a 5% FDR and fold-change cutoff (Fig. 3b). The list of all the transcripts that are DE across BSp, GTPs and combination (BSp + GTPs) treatment groups is displayed in Supplementary File 1: Table S1–S5. The top 30 down-regulated and up-regulated genes ranked by statistical significance with the combination treatment are shown in Tables 1 and 2, respectively. Additionally, out of 146 DEGs in the BSp treatment group and 895 genes in the combination (BSp + GTPs) diet group, we identified 125 overlapping transcripts that were DE. Out of 125 overlapping DEGs, 75 genes were up-regulated, and 50 genes were down-regulated.

**Table 1:** Top 30 differentially expressed down-regulated genes in the combination (BSp 851 + GTPs) treatment group.

Gene symbol	Gene expression fold-change (log_2_FC) (combination vs control)	Average differential expression (combination vs control)	p alue for differential expression (combination vs control)	False discovery rate (FDR)
Capn6	− 2.694	2.719	5.37E−03	0.091
Tmsb15b2	− 1.945	1.183	5.83E−03	0.096
Gm49369	− 1.220	1.608	1.62E−04	0.019
Gm2244	− 1.128	− 0.003	5.54E−03	0.093
Lyz2	− 1.120	7.653	5.64E−03	0.094
Dnajc22	− 1.090	3.272	3.97E−03	0.077
Rps29	− 0.992	7.897	6.08E−03	0.097
Ankle1	− 0.971	1.731	3.14E−03	0.069
Rhox8	− 0.952	0.393	6.16E−03	0.098
Tmsb10	− 0.870	7.369	3.30E−03	0.070
Cox7a1	− 0.855	2.836	4.94E−03	0.088
Tmem254a	− 0.852	2.386	4.44E−03	0.081
Rps12	− 0.822	4.798	1.01E−03	0.038
Dbi	− 0.802	7.809	4.15E−04	0.028
Gnaz	− 0.799	1.881	4.23E−04	0.029
Mxd3	− 0.767	2.716	1.70E−03	0.049
Uqcr10	− 0.749	6.305	4.96E−04	0.030
Ankrd39	− 0.742	2.277	5.28E−03	0.091
Bcl2l11	− 0.730	5.665	8.96E−04	0.037
Rpl41	− 0.729	9.505	1.32E−03	0.043
Atox1	− 0.716	5.389	3.21E−04	0.025
Atp5e	− 0.710	7.092	1.03E−03	0.039
Fkbp11	− 0.696	5.011	3.85E−03	0.077
Smim22	− 0.682	4.657	6.17E−04	0.032
Ppox	− 0.679	2.813	3.42E−03	0.072
Dpm3	− 0.673	4.153	3.46E−03	0.073
Uqcrq	− 0.665	5.657	3.48E−03	0.073
Rnaseh2c	− 0.665	4.686	1.12E−04	0.016
Mtmr12	− 0.665	4.141	4.76E−04	0.030
Cox7c	− 0.657	7.204	3.84E−03	0.077

**Table 2:** Top 30 differentially expressed up-regulated genes in the combination (BSp + GTPs) treatment group.

Gene symbol	Gene expression fold- change (log_2_FC) (combination vs control)	Average differential expression (combination vs control)	p value for differential expression (combination vs control)	False discovery rate (FDR)
Pdx1	4.119	− 2.107	6.03E−03	0.097
Mup14	3.912	− 3.732	3.76E−03	0.076
Adcy10	3.810	− 3.062	4.28E−04	0.029
Carmil3	3.057	− 4.339	4.77E−03	0.086
Muc5b	2.939	− 4.363	5.78E−04	0.032
Fcrla	2.689	− 3.964	1.86E−03	0.052
Slc25a41	2.590	− 2.730	1.29E−03	0.043
Olfr1457	2.555	− 3.260	1.62E−03	0.048
Ptgdr2	2.547	− 5.088	1.94E−04	0.021
Cyp4a31	2.421	− 4.049	2.40E−03	0.060
Efhc2	2.415	− 5.170	3.59E−05	0.009
Olfr1161	2.303	− 3.764	5.38E−03	0.091
Ms4a2	2.222	− 0.630	2.76E−05	0.008
Kcnk10	2.168	− 3.500	1.87E−03	0.052
Tssk2	2.116	− 3.838	3.74E−03	0.076
Olfr316	2.114	− 3.182	5.90E−03	0.096
Dlec1	2.114	− 0.934	4.03E−03	0.078
Olfr1199	2.077	− 3.849	2.70E−03	0.064
Csta1	2.074	− 2.646	2.44E−03	0.060
Ercc6l2	1.773	0.747	8.49E−04	0.036
Palm2	1.725	0.482	2.89E−03	0.066
Olfr263	1.715	− 1.339	1.94E−04	0.021
Angptl8	1.660	− 1.522	1.66E−03	0.049
Bend7	1.420	1.321	6.93E−04	0.033
Pate13	1.402	− 1.125	3.64E−04	0.027
Tmem132d	1.336	− 0.487	2.70E−04	0.024
Slc6a5	1.317	− 2.108	9.75E−04	0.038
Btbd16	1.296	− 0.665	3.10E−04	0.025
Cd55b	1.203	− 0.670	3.82E−03	0.077
Gm26992	1.192	3.332	2.86E−05	0.08

To further elucidate the transcriptional profiles changes across different dietary treatments, we generated a heatmap with the top 30 up-regulated and down-regulated genes in the combination group between rows corresponding to DE genes and columns corresponding to biological replicates in the control and combination treatment groups (Fig. 3c). We also generated a heatmap of DE genes that were up-regulated and downregulated in the BSp treatment group (Supplementary Fig. S3). In the combination treatment group, *Pkd1* and *Bdp1* genes were the top two up-regulated genes. These genes are found to be down-regulated in many different types of cancers. *Pkd1* is a tumor suppressor gene that plays a crucial role in many different types of cancers including BC. *Bdp1* is a protein coding gene found in TFIIIB that can regulate JNK1 expression in c-jun N-terminal kinases (JNKs), which displays both oncogenic and tumor suppressor properties^12,13^. Furthermore, the most down-regulated genes were related to biological processes such as DNA-templated transcription positive regulation of “DNA binding” “protein phosphorylation”, “intracellular signal transduction” and top up-regulated genes were related to “histone acetylation”, “covalent chromatin modifications”, “apoptosis” and “DNA-methylation”.

**Figure 4:**
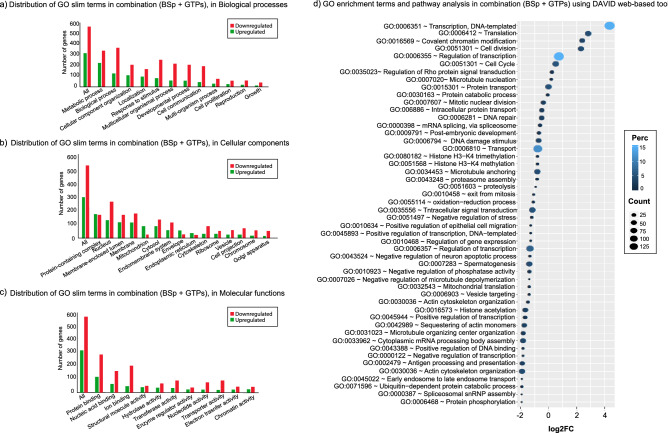
Bar plot distribution of GO slim terms of differentially expressed transcripts related to the combination (BSp + GTPs) treatment group in (**a**) biological process, (**b**) cellular components and (**c**) molecular functions wherein red bars represent downregulated genes and green bars represent up-regulated genes. The height in the bar plot represents the total number of differentially expressed genes. (**d**) Gene ontology enrichment terms and REACTOME pathways analyses using DAVID web-based tool. The plot is sorted based on decreasing FC wherein Y-axis represents specific GO terms related to biological pathways and X-axis represents log_10_(FC) associated with each GO term.

To gain a holistic understanding of DEGs with combination and BSp treatments, we used WebGestalt software to perform GO SLIM subset analyses which can identify major clusters within various biological processes, cellular components and molecular functions. In the combination treatment group, among the biological processes category, the major subsets were “metabolic processes” and “biological response to stimulus” (Fig. 4a). Similarly, in the cellular component category, the most abundant terms were “membrane’, “nucleus” and “cytosol” (Fig. 4b) and in the molecular function category and the most frequent terms were related to “protein binding”, “ion binding” and “nucleotide binding” (Fig. 4c). Due to lesser transcripts that were DE in the BSp treatment group, we noticed fewer abundance at these subcategories level (Supplementary Fig. S4a–c).

Additionally, we used DAVID to perform a broader analysis of specific biological function related to DEGs in BSp and combination treatment groups. In the BSp treatment group, 33 GO terms (p value ≤ 0.05) mainly related to regulation of transcription” (GO:0006355 and GO:0006351), “oxidative-reduction process” (GO: 0055114) and “DNA methylation” (GO:0006306) (Supplementary Fig. S4d). Unlike the BSp treatment group, combination treatment group exhibited higher gene ontology functions with 50 GO terms which were statistically significant DE transcripts. These transcripts were primarily related to various epigenetics mechanisms and other biological pathways such as “cellular response to DNA damage stimulus” (GO: 0006974), “covalent chromatin modifications” (GO:0016569), “DNA repair” (GO:0006281), “mitotic nuclear division” (GO: 0007067), “methylation” (GO:00032259), “histone H3-K4 methylation” (GO:0051568) and “histone H3-K4 trimethylation” (GO:0080182) (Fig. 4d). Although the combination treatment group exhibited more associations with gene ontology functions, there were many biological processes overlapping between the treatment groups.

Based on these gene expression results, we concluded that the combination (BSp + GTPs) treatment can impose greater effects on expression level changes by identifying a greater number of DE genes (DEG_Combination_ = 895) in comparison to BSp and GTPs administered alone with less DE genes (DEG_BSp_ = 145 and DEG_GTPs =_ 0). Further, these transcripts in the combination treatment group elicited greater changes at the functional level by identifying various epigenetics modifications which might eventually result in delaying or inhibiting ER(−) mammary cancer. Consistent with the phenotypic changes that the combination treatment can lead to the most promising inhibitory effects on mammary cancer development as compared to single compound treatment alone, these dramatic transcriptomic pattern changes may contribute to chemopreventive effects induced by combination treatment against mammary cancer.

### Combination of dietary botanicals induces genome-wide differential DNA methylation patterns

To further assess the changes in DNA methylation patterns on combinatorial dietary treatment in comparison to BSp and GTPs treatment alone, we applied RRBS analyses across harvested mammary tumor samples. A total of twenty libraries were constructed, and each of them produced a minimum of 5 Gb clean reads, which were sequenced and aligned to the reference genome of *Mus musculus* (mm10) using Bismark (https://www.bioinformatics.babraham.ac.uk/projects/bismark/). The reads of individual samples mapped to the reference genome, thereby producing the relevant BAM files for different samples within each group. Furthermore, these files were used for final downstream analyses resulting in CpGs methylation levels for each treatment group (5 samples/treatment group). Additionally, we identified differentially methylated regions (DMRs) within a minimum range of 500 bp across different treatment groups. We performed empirical optimization of the methylation regions generated using Bismark across different treatment groups. Additionally, we performed dependency adjustment for DMRs within each treatment group and identified statistically significant differentially methylated genes (DMGs) in the BSp, GTPs and combination treatment group.

**Figure 5:**
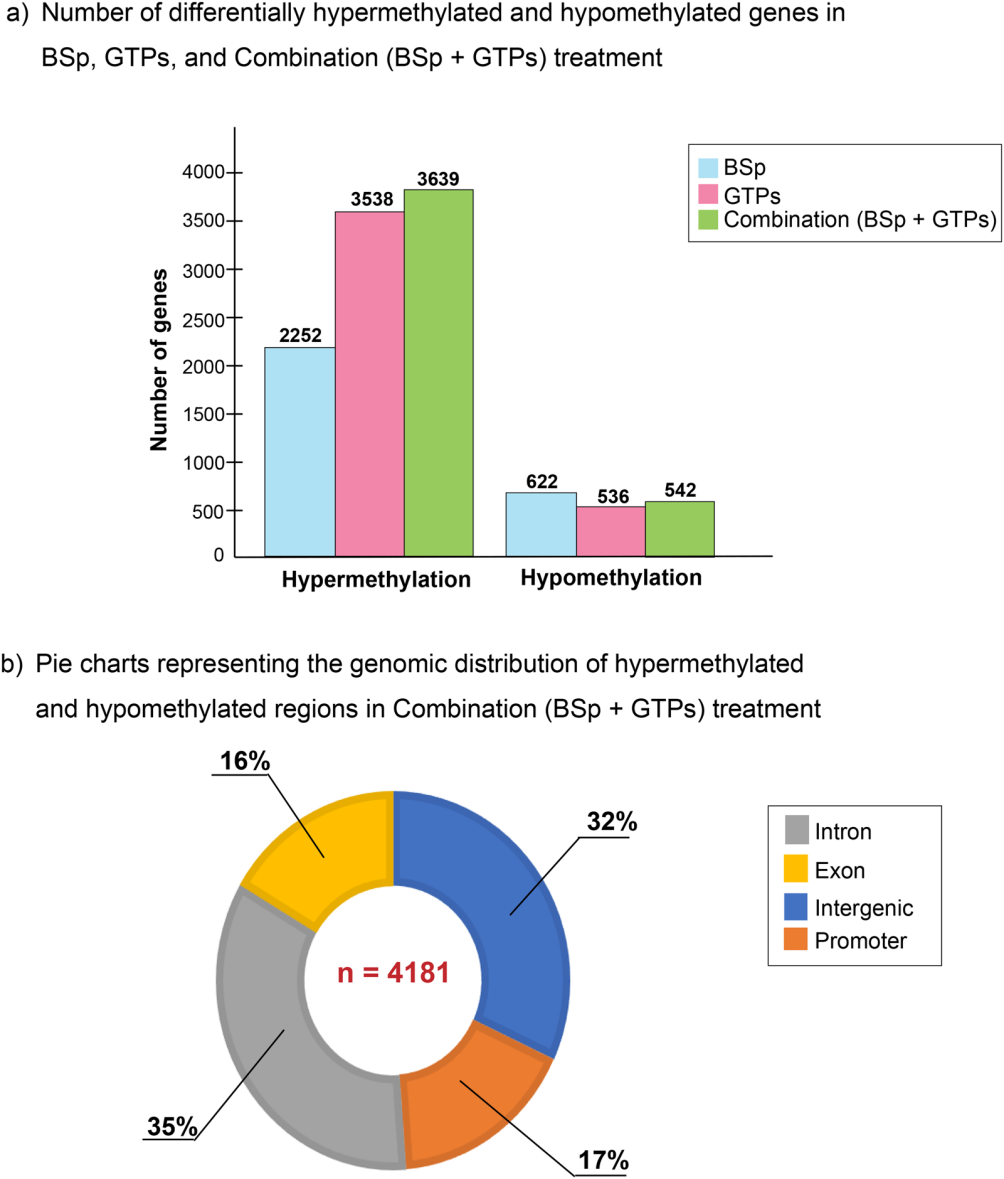
Differential DNA methylation analyses by RRBS across different treatment groups. (**a**) Bar plot representing different numbers of differentially hypomethylated and hypermethylated genes in BSp, GTPs and the combination (BSp + GTPs) treatment groups. The height in the bar plot represents the total number of differentially methylated genes in BSp (blue color), GTPs (pink color) and the combination (green color) treatment groups. (**b**) Pie charts representing the genomic distribution of hypo- and hypermethylated regions in the combination treatment (N_DMRs_ = 4181) group. Grey color represents Intronic regions (35%), yellow color represents exonic regions (16%), blue color represents intergenic regions (32%) and orange color represents promoter regions (17%).

In the BSp and GTPs groups, a total of 2874 genes and 4074 genes associated with DMGs were identified (p value ≤ 0.05), respectively. Out of 2874 genes in the BSp group, 622 genes were hypomethylated and 2252 genes were hypermethylated, and out of 4074 genes in the GTPs group, 536 genes were hypomethylated and 3538 genes were hypermethylated (Fig. 5a). Comprehensive lists of all the transcripts that are DM in the different treatment groups are provided in Supplementary File 2: Tables S6–S12. Based on the annotation results, the DMRs in the BSp treatment group were mainly distributed in the intronic regions (35%), exonic regions (18%), intergenic regions (31%) and promoter regions (16%) (Supplementary Fig. S5a). The DMRs in the GTPs treatment group were found in the intronic regions (38%), exonic regions (18%), intergenic regions (32%) and promoter regions (17%) (Supplementary Fig. S5b). Unlike BSp and GTPs treatments administered individually, the combination treatment exhibited greater variation at the methylation level with a total of 4181 genes associated with DMGs. Out of 4181 genes in the combination treatment group, 542 genes were hypomethylated and 3639 genes were hypermethylated (Fig. 5a). In the combination treatment group, the DMRs were distributed in the intronic regions (35%), exonic regions (16%), intergenic regions (32%) and promoter regions (17%) (Fig. 5b).

This list served (Supplementary File 2: Table S10) as a reference list for identification of DMRs in the combination treatment group and was further used for correlation with transcripts that were DE in the combination treatment group.

### Correlative analyses of DNA methylation and gene transcription

To further elucidate the potential role of DNA methylation on gene expression, we correlated DEGs obtained from RNA-seq and DMGs obtained from RRBS and identified significant overlapping genes in different treatment groups. In the BSp treatment group, a total number of 146 DEGs and 1435 DMGs were identified, amongst which only 8 DEGs overlapped with DMGs (*Bend3*, *Cox7a2l*, *Eml4*, *Atp5e*, *Flrt3*, *Rps5*, *Ppp1515b* and *Cdc42bpb*) (Supplementary Fig. S6a). The correlation of DMGs with DEGs was not performed in the GTPs treatment group as there we no DEGs identified in GTPs treatment group. Subsequently, a heatmap between DM and DE in BSp treatment group was generated in order to visualize the transcription and methylation level changes between these overlapping genes (Supplementary Fig. S6b). Out of 8 genes which were DM and DE, 4 genes were up-regulated and hypomethylated and 4 genes were down-regulated and hypermethylated (Supplementary File 2: Table S8).

**Figure 6:**
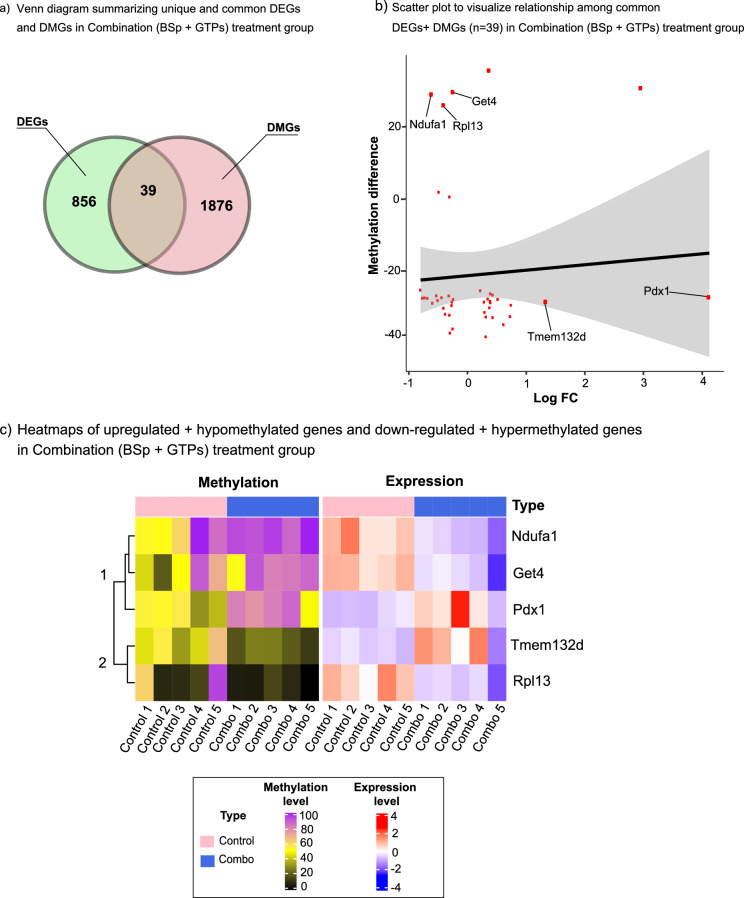
Correlation of DEGs and DMGs in the combination treatment group. (**a**) Venn diagram representing unique and overlapping differentially expressed genes (DEGs) and differentially methylated genes (DMGs) in the combination (BSp + GTPs) treatment group. (**b**) Scatter plot for 39 genes which are differentially expressed and methylated. The y-axis represents the methylation difference across 39 genes (dots in red color) and x-axis represents log_10_FC. Out of 39 transcripts, 2 genes (*Tmem132d* and *Pdx1*) were up-regulated and hypomethylated and 3 genes (*Ndufa1*, *Rpl13* and *Get4*) were downregulated and hypermethylated. (**c**) Heatmap representing overlapping 5 up- and downregulated genes in the combination (BSp + GTPs) treatment group based on q value. Each row corresponds to differentially expressed and differentially methylated transcripts and each column represents biological replicates in control (N = 5) and the combination (N = 5) treatment group. Blue color denotes lower expression levels and red color denotes higher expression levels.

Unlike the BSp treatment group, the combination treatment group exhibited a higher correlation among DEGs and DMGs. In the combination treatment group, out of 895 DEGs with 575 up-regulated genes and 320 down-regulated genes, and 4181 DMRs with 542 hypomethylated and 3639 hypermethylated regions, we identified 39 overlapping genes (Fig. 6a). Table 3 provides a comprehensive depiction of overlapping genes in the combination treatment group which were DE and DM simultaneously. Subsequently, 2 genes (*Pdx1* and *Tmem132d*) were identified with a positive association of up-regulated DEGs and hypomethylated genes, while 3 genes (*Get4, Rpl13* and *Ndufa1*) were found to be down-regulated DEGs and hypermethylated genes. To better visualize the relationship between the 5 transcripts that were DM (meth.diff) and DE, we generated a scatter plot between methylation difference and FC (Fig. 6b). The heatmap in Fig. 6c shows DE and DM between rows and columns corresponding to biological replicates in the combination treatment group. Table 4 provides a reference list of unique transcripts that showed significantly differential changes (p value ≤ 0.05) with positive correlation of gene expression and DNA methylation patterns (up-regulated gene expression with hypomethylated DNA and down-regulated gene expression with DNA hypermethylated) in the combination treatment group.

**Table 3:** Overlapping DEGs with expression level changes and DMGs with methylation level changes in the combination (BSp + GTPs) treatment group.

Gene symbol	Gene expression fold-change (log_2_FC) (combination vs control)	Average differential expression (combination vs control)	p value for differential expression (combination vs control)	Chromosomal location	Methylation difference (combination vs control)
Aff1	0.432	6.965	3.74E−05	5	− 32.812
Ap1s1	− 0.239	6.395	1.14E−03	5	− 27.815
Ap2s1	− 0.377	6.280	3.02E−04	7	− 27.403
Cdc42bpb	0.349	6.548	3.50E−04	12	− 25.862
Cdk13	0.222	5.982	4.33E−03	13	− 25.380
Clasp2	0.393	5.961	1.72E−03	9	− 26.242
Etl4	0.743	6.713	3.01E−04	2	− 25.050
Get4	− 0.251	5.785	5.27E−03	5	29.204
Gm17018	− 0.262	4.498	3.76E−03	19	− 28.522
Gnaz	− 0.799	1.881	4.23E−04	10	− 25.221
Hsbp1	− 0.303	6.770	3.06E−03	8	− 26.257
Iffo2	0.355	4.731	3.89E−03	4	40.880
Il15ra	0.517	4.863	6.00E−03	2	− 27.794
Kdm2b	0.294	5.574	4.60E−03	5	− 26.947
Mrps12	− 0.295	4.972	2.78E−04	7	− 38.379
Muc5b	2.939	− 4.363	5.78E−04	7	30.468
Mxd3	− 0.767	2.716	1.70E−03	13	− 27.448
Naaladl2	0.728	4.932	6.85E−04	3	− 32.540
Ndufa1	− 0.606	4.841	2.85E−03	X	28.913
Nr2c2	0.431	4.959	1.35E−03	6	− 26.573
Pard3	0.315	6.086	1.18E−03	8	− 31.251
Pdx1	4.119	− 2.107	6.03E−03	5	− 27.076
Phb	− 0.314	7.148	2.80E−03	11	− 26.850
Pomp	− 0.303	7.168	1.40E−03	5	27.013
Prex2	0.616	2.794	5.10E−03	1	− 34.783
Psmb3	− 0.504	6.188	2.35E−03	11	− 28.077
Psmb6	− 0.437	6.948	1.80E−03	11	− 27.334
Psmd9	− 0.248	4.914	3.66E−03	5	− 27.477
Rbx1	− 0.487	6.285	8.75E−04	15	− 25.898
Rpl13	− 0.399	9.816	3.37E−04	8	25.913
Rpl41	− 0.729	9.505	1.32E−03	10	− 27.371
Rps5	− 0.406	9.342	9.02E−05	7	− 26.124
Selenow	− 0.267	6.793	6.07E−03	7	− 29.497
Smim22	− 0.682	4.657	6.17E−04	16	− 27.510
Tmem184a	0.284	6.827	5.77E−03	5	− 29.260
Tnks	0.317	6.279	9.09E−04	8	− 33.032
Trim56	0.378	6.431	1.79E−04	5	− 30.123
Use1	− 0.527	5.689	2.76E−03	8	− 26.789
Tmem132d	1.336	− 0.487	2.70E−04	5	− 31.003

**Table 4:** Target genes with positive correlation with gene expression and DNA 860 methylation changes in the combination (BSp + GTPs) treatment group.

Gene symbol	Gene expression fold-change (log_2_FC) (combination vs control)	Average differential expression (combination vs control)	p value for differential expression (combination vs control)	Chromosomal location	Methylation difference (combination vs control)
Get4	− 0.251	5.785	5.27E−03	5	29.204
Ndufa1	− 0.606	4.841	2.85E−03	X	28.913
Pdx1	4.119	− 2.107	6.03E−03	5	− 27.076
Rpl13	− 0.399	9.816	3.37E−04	8	25.913
Tmem132d	1.336	− 0.487	2.70E−04	5	− 31.003

A pathway enrichment analyses using ConsensusPathDB was performed to explore the pathways associated with the overlapping genes. The up-regulated and hypomethylated genes were enriched for “transcription-related”, “DNA binding”, “regulation of gene expression” and “cell division” pathway. The down-regulated and hypermethylated genes were enriched with “apoptosis”, “oxidative-phosphorylation”, “translation” and “DNA-methylation” pathway (Table 5). These results suggest that the molecular mechanisms that reinforce the biologically beneficial effects of lifestyle modification by consumption of BSp + GTPs together drive transcriptome level changes significantly and have a substantial effect on methylation level changes.

**Table 5:** Pathway enrichment analyses of overlapped genes between RRBS and RNA-seq data in the combination (BSp + GTPs) treatment group.

Gene symbol	Pathways related to gene symbol	p value for differential expression (combination vs control)
**Overlapping up-regulated DEGs and hypomethylated DMGs**		
Pdx1	Transcription, DNA-templated	2.57E−08
Pdx1	Negative regulation of transcription from RNA polymerase II promoter	8.58E−07
Pdx1	Positive regulation of DNA binding	2.85E−06
Tmem132d	Negative regulation of phosphatase activity	1.80E−04
Tmem132d	Regulation of gene expression	3.34E−04
Pdx1	Cell division	4.38E−04
**Overlapping down-regulated DEGs and hypermethylated DMGs**		
Ndufa1	Apoptosis	5.06E−04
Ndufa1	Oxidation-phosphorylation process	5.74E−04
Rpl13	Translation	5.80E−04
Get4	DNA methylation	6.54E−04

### Validation of target genes with unique differentially expressed (DE) and differentially methylation (DM) patterns in combination treatment by qRT-PCR

**Figure 7:**
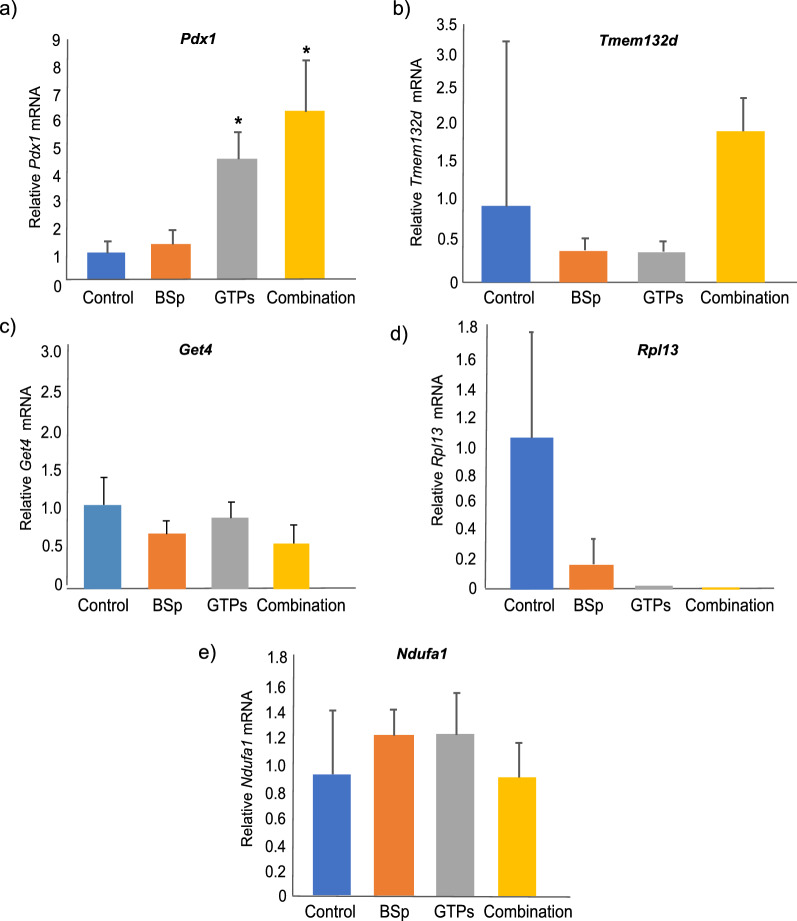
Validation of unique differentially expressed and differentially methylated genes in combination (BSp + GTPs) treatment group using quantitative real-time PCR to measure relative expression of (**a**) *Pdx1* (**b**) *Tmem132d* (**c**) *Get4* (**d**) *Rpl13* and (**e**) *Ndufa1* in BSp, GTPs and the combination (BSp + GTPs) treatment groups. The experiments were performed in triplicate from three independent experiments and further normalized to internal control and calibrated to levels in control (untreated) samples. Columns mean; bars, standard error. *p value < 0.05.

To further verify the identified target genes that showed significant changes in DE and DM patterns in response to the combination treatment, we evaluated the expression of *Pdx1*, *Tmem132d, Get4, Rpl13* and *Ndufa1* genes in mouse mammary tumors using qRT-PCR. We found that combinatorial treatment induced different mRNA expression levels in all tested genes as shown in Fig. 7a–e. Studies have revealed that amongst the 5 genes, *Pdx1*^14^ and *Tmem132d*^15^ are tumor suppressor genes and *Get4*^16^*, Rpl13*^17^ and *Ndufa1*^18^ are tumor promoting genes. *Pdx1* and *Tmem132d* were up-regulated and hypomethylated in genome-wide analysis and the results of qRT-PCR revealed the relative expression of these genes were overexpressed in combination treatment group (*Pdx1*_Combination_ = 6.27 and *Tmem132d*_Combination_ = 2.00) in comparison to BSp group (*Pdx1*_BSp_ = 1.34 and *Tmem132d*_BSp_ = 0.43) and GTPs group (*Pdx1*_GTPs_ = 4.50 and *Tmem132d*_GTPs_ = 0.40) alone (Fig. 7a,b). Subsequently, the results of qRT-PCR in *Get4, Rpl13* and *Ndufa1* displayed the lowest relative expression in the combination treatment group (*Get4*Combination = 0.54*, Rpl13*Combination = 1.71E−05 and *Ndufa1*Combination = 0.98) in comparison to BSp group (*Get4*_BSp_ = 0.65*, Rpl13*_BSp_ = 0.17 and *Ndufa1*_BSp_ = 1.34) and GTPs group (*Get4*_GTPs_ = 0.84*, Rpl13*_GTPs_ = 3.19E−05 and *Ndufa1*_GTPs_ = 1.35) alone (Fig. 7c–e). Consistent with our genome-wide analysis, these results indicate that combined BSp + GTPs treatment are highly effective in inhibiting early BC in comparison to BSp treatment group and GTPs treatment group alone, which may contribute to epigenetic regulation of these key genes.

## Discussion

Novel therapeutic approaches and targets have been developed for different types of cancer^19^. For instance, a recent study on molecular signatures identified various mutant proteins related to BC such as BRCA1, BRCA2 and PTEN. These molecular signatures can potentially serve as a potential target for understanding various changes at the genomic level during breast tumor progression^20^. Significant evidence indicates the role of various dietary phytochemicals or compounds in cancer prevention by elucidating changes at the molecular and biological levels^21,22^. Previous studies have also suggested that several mechanisms may lead to various preventive and therapeutic effects of dietary botanicals on different types of cancers such as apoptosis^23^, cell cycle arrest^24^ and regulation of various signaling pathways such as MYC-WWP1 inhibitory pathway targeting PTEN^25^.

In our previous studies, we found that BSp^26,27^ and GTPs^28,29^ can potentially act as therapeutic and preventive agents against BC. However, the molecular mechanisms through which these dietary botanicals (BSp and GTPs alone and in combination) affect different stages of life remain unknown.

In this study, we evaluated the effect of BSp and GTPs alone and in combination in prevention of ER(−) mammary cancer. Herein, we administered BSp, GTPs and a combination of BSp and GTPs to transgenic Her2/neu mice. As a result, the combination treatment group showed stronger efficacy towards inhibiting tumor growth. Furthermore, the tumor incidence rates demonstrated a significant decrease in the BSp and combination treatment groups along with a decline in tumor weight. To better understand the underlying mechanisms behind the impacts of these dietary botanicals on BC prevention or delay, we characterized the global DNA methylation and gene expression patterns in mammary tumors in response to BSp, GTPs or in their combination (BSp + GTPs). This is the first report describing the impacts of BSp, GTPs or combination (BSp + GTPs) dietary treatment at both transcriptomic and methylomic levels in mammary tumors.

Although high throughput analyses revealed various changes across different treatment groups, the combinatorial administration has a greater affect in comparison to BSp and GTPs administered individually. Gene set enrichment analyses identified different epigenetics pathways that were modulated due to these dietary treatments.

Firstly, we identified 145 DEGs and 895 DEGs that were obtained in response to the BSp treatment group or the combination treatment, respectively and no DEGs in GTPs treatment group. We identified DEGs between the control and the GTPs group, as well as other treatment groups, with the lower FDR-adjusted p value of 0.1 and higher fold change (higher than 2, in either direction). Although no DEGs were screened out from the GTPs group, numerous genes were up- or down-regulated with a fold-change less than 2 (Supplementary File 1: Table S6). Thus, significantly lower tumor biomass in GTPs group should be primarily attribute to an accumulative effect of those changed expressed genes. Additionally, posttranscriptional and posttranslational regulation, like noncoding RNA and histone modification, are proven to remarkably affect protein expression, leading to efficient regulation of biological processes, without noticeable changes at the transcriptional level. Future studies are warranted to investigate underlying mechanisms of mammary tumor suppression due to our dietary treatments at different regulatory levels. Subsequently, we identified a group of target genes (such as *Pdx1*, *Tmem132d*, *Get4*, *Ndufa1* and *Rpl13*) that could be regulated by epigenetic mechanisms such as DNA methylation in the combination treatment group. Based on our results from DNA methylation profiles, the breast tumors treated with the combination diet (BSp + GTPs) tend to be more hypermethylated compared to the control group, which provides strong evidence that most of the CpGs are unmethylated in the control group. These DNA modifications at the global level may lead to changes in gene expression that are related to upregulation and down-regulation. Based on our analyses, we found considerably less hypomethylated regions in comparison to hypermethylated regions across different dietary treatment groups (Combination_hypo_ = 542 and Combination_hyper_ = 3639). Subsequently, in our previous studies, we also found that dietary BSp treatment potentially increased DNA methylation levels globally^11^.

We identified several target genes that show positive correlation of gene expression and DNA methylation changes through integrated analyses (Table 4). For example, the combination treatment with BSp and GTPs up-regulated gene expression of *Pdx1* and *Tmem132d* genes. Pancreatic and duodenal homeobox 1 (*Pdx1*) is a transcription factor expressed in the promoter regions of *Mus musculus* and human olfactory epithelium (MOE). *Pdx1* primarily contributes to morphogenesis during the development of mouse embryonic pancreas^30^. A plethora of studies have suggested that *Pdx1* is a tumor suppressor gene which is primarily involved in different types of cancers such as BC, pancreatic cancer, gastric cancer and pseudopapillary tumors^31–33^. Further, we found that the *Pdx1* gene was up-regulated and hypomethylated after administration of the combination (BSp + GTPs) treatment. However, *Pdx1* was neither DE or DM after the administration of BSp or GTPs treatment alone. This could potentially be due to a synergistic effect in combination treatment in comparison to BSp or GTPs alone. Similarly, transmembrane protein 132d (*Tmem132d*) is a tumor suppressor gene actively involved in different types of cancer such as lung cancer^15^, ovarian cancer^34^ and BC^35^. *Tmem132d* was up-regulated and hypomethylated in the combination treatment with BSp and GTPs but not in BSp or GTPs treatment alone. These gene expressions were found to be positively correlated with their DNA methylation changes with a methylation difference of − 27.08 and − 31.00. Functional annotations of these genes identified key pathways that were primarily related to “DNAtemplated transcription”, “positive regulation of DNA binding”, “regulation of gene expression” and “cell division”.

In addition, our analyses also revealed that combinatorial administration of BSp and GTPs resulted in down-regulated and hypermethylated changes in the *Ndufa1*, *Rpl13* and *Get4* genes. The *Ndufa1* gene (MWFE protein) is the first component of the electron transport chain that accepts electrons from NADH oxidation^36^. It is primarily responsible for NADH-ubiquinone oxidoreductase (complex-I)^37–39^. This gene is also responsible for direct effects on reactive oxygen species (ROS)^39^, functional complex-I assembly^40^ and possess a wide variety of roles in aerobic activity^41^. These functionalities associated with *Ndufa1* eventually play a significant role in genetic instability and tumorigenesis. In our analyses, we identified *Ndufa1* to be downregulated and hypermethylated with a methylation difference of 28.91. Another gene, *Rpl13* was found to be down-regulated in our study. Studies have shown *Rpl13* plays a crucial role in tumor development in gastrointestinal carcinoma^42^, colorectal cancer^43^, prostate cancer and BC^44^ with greater proliferative capacity and attenuated chemoresistance^45^. Additionally, functional ontology of these down-regulated genes revealed pathways that were primarily associated with various biological pathways such as “apoptosis”, “oxidative-phosphorylation process”, “DNA methylation activity” and “translation related pathway”. Furthermore, a study in colorectal cancer reported that Golgi to ER traffic protein 6 (*Get4*) has been considered to promote tumorigenesis or tumor progression by demonstrating clinical significance of *Get4* expression in colorectal cancer^16^.

Subsequently, we validated the gene expression of these uniquely identified target genes by real time RT-PCR assay. Our results indicated that the combinatorial treatment with BSp and GTPs can induce significant gene expression in most of the tested genes, which are also consistent with our RNA-seq and RRBS results. Thus, combinatorial treatment with BSp and GTPs may induce epigenetic regulations of these key tumor-related genes with important biological functions, which may contribute to its tumor inhibitory effects on mammary cancer development consistent with the cancer phenotypic results that we have observed in this study.

Altogether, these findings, based on an unbiased analysis of DE and DM genes along with functional characterization, identified that combined administration of BSp and GTPs dietary botanicals have shown greater impact on tumor suppression, gene expression and DNA methylation levels in comparison to BSp or GTPs treatment alone. We posit that the alteration in gene expression coupled with methylation changes modulates epigenetics pathways to a greater extent with the combinatorial treatment. Overall, our results indicate that the combination treatment consisting of BSp and GTPs may reverse epigenetic aberrations in BC leading to beneficial outcomes such as slow progression or delay of breast tumorigenesis. Therefore, these latter results could facilitate further understanding of the underlying etiology behind these epigenetic alterations.

## Conclusion

In summary, we found DE and DM genes across different treatment groups and detected a subset of correlated genes that were DE and DM by using an unbiased approach. Additionally, we show that in comparison to BSp or GTPs administered alone, combinatorial treatment consisting of BSp and GTPs has a greater impact on transcriptomic and methylomic changes that may contribute towards reducing the tumor incidence rate of ER(−) BC consistent with the cancer phenotypic observations in this study. Identifying these key genes that potentially contribute towards BC-associated risk factors could serve as a key avenue in the area of translational precision medicine and eventually lead to improving risk assessment of BC. Further, the functional characterization of these transcripts may lead to the identification of novel therapeutic strategies against ER(−) BC.

## Materials and methods

### Animals

The animal study was reviewed, approved and performed in accordance with relevant guidelines and regulations by Institutional Animal Use and Care Committee of the University of Alabama at Birmingham (IACUC; Animal Project Numbers: 10088 and 20653). Spontaneous mammary tumors in female wildtype Her2/neu [FVB-Tg (MMTVErbb2) NK1Mul/J] mice were used as a preclinical model for ER(−) BC. Previous studies have shown that Her2/neu mice develop ER(−) mammary tumors at early age of approximately 20 weeks with median latency around 30 weeks^46^. In order to generate experimental colonies, the breeder mice at 4 weeks of age were obtained from the Jackson Laboratory (Bar Harbor, ME) and were bred from 10 weeks of age. The pups were weaned at 21 days after birth and were further tagged and genotyped, and a standard PCR analyses was performed on the tail of mice^47^. Mice were contained in the Animal Resource Facility of the University of Alabama at Birmingham and were sustained under 12 h dark/12 h light cycle, 24 ± 2 °C temperatures and 50 ± 10% humidity. To determine the power and sample size, we used an online Power and Sample Size Calculator based on 2-proportion comparison (http://powerandsamplesize.com/).

### Mice dietary treatment

Female Her2/neu mice were segregated into four treatment groups (N_Control_ = 20, N_BSp_ = 20, N_GTPs_ = 20 and N_Combination_ = 20) and fed with dietary botanicals from prepubescence (3 weeks) until termination (31 weeks). The dietary botanicals were stored in sealed containers and refrigerated (2 °C for 6 months and − 20 °C for longer term)^48^. Out of 20 mice in each treatment group, 5 mice from each treatment group were randomly chosen for transcriptomic and methylomic analyses. The first group was comprised of control mice (N_Control_ = 5) which were administered control AIN-93G diet. In group 2 (N_BSp_ = 5), mice were fed with SFNrich broccoli sprouts powder (Natural Sprout Co.) which was added at 26% (w/w) into a modified AIN93G diet pellet from TestDiet, St. Louis, MO. 5.13–6.60 μM SFN per gram was evaluated for each batch of broccoli sprouts powder. In group 3 (N_GTPs_ = 5), mice were orally fed with GTPs Sunphenon 90D (Sunphenon 90D, Taiyo International, Inc., Minneapolis, Minnesota) which was added at 0.5% in drinking water. Sunphenon 90D (Taiyo Inc., Minneapolis, MN, USA) is comprised of > 90% polyphenols, > 80% catechins, > 45% EGCG and < 1% caffeine, and is a decaffeinated extract of green tea encompassing purified polyphenols rich in green tea catechins. In group 4 (N_Combination_ = 5), mice were fed with a combination diet of both BSp diet (Group 2) in pellets and GTPs (Group 3). Treatments were continued throughout the study until termination. Subsequently, the mice mammary tumor tissues were collected at 31 weeks during termination.

### Tumor observation and sampling

The female Her2/neu WT mice model developed mammary tumors at an early age at around 20 weeks. Tumor incidence was measured and recorded weekly for tumor development throughout the experiment and the experiment was terminated when the average tumor diameter of mice in the control group exceeded 1.0 cm. Mouse body weight was recorded biweekly from 4 to 28 wk of age. Mouse food and water intakes were measured at 4,12, 20 and 28 wk of age respectively. At the end of the experiment, mice were euthanized by CO_2_ and the mammary tumors were excised, weighed and further stored in liquid nitrogen for subsequent analysis.

### Experimental design

The harvested tumor samples derived from 20 Her2/neu mice for different treatment groups were used for an overall construction 20 RNA-seq libraries and 20 RRBS methylation libraries. The complete workflow based on the employed approach for experimental design and bioinformatics analyses of RNA-seq and RRBS data to generate transcript level abundance and genome-wide DNA methylation profile for different phytochemical treatments is graphically demonstrated in Fig. 2. The details of the analytical steps related to analyses are further described in the sections with various subparts.

### DNA and RNA extraction

Genomic DNA (gDNA) was extracted from frozen mammary tumor tissues of mice using the DNAeasy kit (Qiagen, Valencia, CA) based on manufacturer’s guidelines. DNA concentration was determined using a NanoDrop spectrophotometer and the DNA quantity was measured using Qubit. Subsequently, total RNA was extracted from frozen mammary tumor tissues of mice using TRIzol reagent (Sigma-Aldrich, St. Louis, MO) based on the manufacturer’s protocol. The RNA concentrations were determined with NanoDrop spectrophotometer and RNA Nano bioanalyzer chip was used to evaluate the integrity of RNA. Only the RNA samples with an integrity number greater than 7 were further used for sequencing.

### Statistical analyses on animal experiments

Power calculation and sample size was measured using one-side 2-proportion comparison using an online power calculator (http://powerandsamplesize.com/). In order to avoid any chances of single false positive during multiple comparison, Bonferroni adjustment with Power = 80%, Significance level = 0.01 and α = 0.05 was performed amongst four different treatment groups and statistical analyses was performed by SPSS software (v24.0). Furthermore, tumor incidence and significance were determined using Chi-square test. Two-tailed student’s t test was performed to compare two treatment groups, and one-way independent ANOVA was performed to compare three or more groups. Tukey’s post-hoc test was performed to determine significance between the groups. Error bars were standard error of the mean obtained from experiments. Statistically significant outcomes were represented as ** for p value < 0.01 and * for p value < 0.05.

### Sequencing, processing and alignment of RNA-seq and RRBS libraries

RRBS pair-end libraries were sequenced on Illumina NextSeq 500 platform. The quality of the raw Fastq were assessed using FastQC (v0.11.4). The adapter sequences were trimmed using trim_galore based on NuGEN Ovation RRBS system. The trimmed reads were aligned to the reference genome for mouse GRCm38/mm10 using Bismark alignment with default parameter settings. As a result, context-dependent methylation (CpG sites) call files were generated using bismark_methylation extractor.

RNA-seq pair-end libraries were sequenced on Illumina NextSeq500. We performed fastQC to check the quality of the raw fastq data per samples. After FastQC, the RNA-Seq fastq reads were aligned to the mouse reference genome GRCm38/mm10 using Kallisto with their default parameter settings. The aligned BAM files were further processed using Kallisto (v 0.43.1-intel-2017a)^49^. A transcription level estimates (or abundance estimate) file was generated for each sample/treatment group.

Further, a transcript-level estimates file was used as an input in tximport package^50^ in R (v3.6.1) and the transcript level information was summarized to gene-level with their respective expression values.

### Identification of differentially methylated (DM) CpG sites and differentially methylated regions (DMRs)

Differential methylation analyses was performed using methylKit^51^ package in R (version 3.6.1). Firstly, the methylation call files generated using Bismark^52^ for treatment groups (BSp, GTPs, BSp + GTPs) were used as input files to generate CpG regions profiles. Subsequently, methylKit was used to identify DMG’s and DMR’s based on the false discovery rate (FDR)  ≤ 0.05. In order to further explore the methylation profiles between the control and the treatment groups (BSp, GTPs and BSp + GTPs), hierarchical clustering was performed in R using hclust package.

### Identification of differentially expressed genes (DEGs)

To characterize the transcriptional level changes in malignant tumors of Her2/neu mice (5 mice per treatment group) after BSp treatment, GTPs treatment, combined GTPs + BSp treatment, and control group (untreated group), we utilized R/Bioconductor package Limma (version 3.6.1). Limma package was used to evaluate differential gene expression by performing quantile normalization wherein an attempt is made to match gene count distributions across the samples in a dataset^53^. Subsequently, the significant threshold of DEGs was set as|log_2_(fold-change)|> 2 and false discovery rate (FDR)  ≤ 0.01.

### Integrated analyses of DNA methylation and gene expression

To further elucidate the potential relationship between DNA methylation and mRNA expression, we correlated CGI-DMRs with their respective DEGs by selecting the significant DMRs-DEGs pair with p value < 0.05. In order to visualize the relationship between DEGs and DMRs, scatterplot with FC was generated using ggplot package in R (version 3.6.1)^54^.

### Gene set function enrichment

Significant transcripts at the transcriptome level and methylome levels (p < 0.05) were considered to perform Gene Ontology (GO) enrichment. To identify associations of genes with GO terms, functional enrichment was conducted using web-based tool DAVID^55^ and gsva package comprised of Reactome and KEGG database in R (version 3.6.1). Furthermore, we used a web-based tool WebGetSalt to perform an over-representation analyses with a significance level of 5% FDR^56^.

### Quantitative real-time PCR (qRT-PCR)

Total RNA was harvested from mouse mammary tissues by using QIAGEN RNeasy Plus Kit. Following extraction following the manufacturer’s protocol. Following the extraction, RNA was reverse transcribed to cDNA by using iScript cDNA synthesis kit (BioRad) according to the manufacturer's instructions^57,58^. Gene expression for specific genes of interest was performed in triplicate and further analyzed using real-time PCR by SYBR GreenER qPCR Supermix (Invitrogen) in a Roche LC480 thermocycler. For the PCR setup, we used 1 µL of cDNA, 5 µL of iTaq SYBR green from Bio-Rad, 2 µL of nuclease-free water, 1 µL of forward and reverse primers for specific genes of interest with total volume of 10 µL. Once the samples were prepared, gene expression was evaluated in triplicates and PCR was conducted with CFX Connect Real Time system (Bio-Rad). Specific gene primers for *Pdx1, Tmem132d, Rpl13, Get4* and *Ndufa1* was obtained from Integrated DNA Technologies (Coralville, IA, USA). The primer sequences used were as follows: 5′-GTGGACAGTGAGGCCAGGAT-3′ (F) and 5′-GATTACTGCTCTGGCTCCTAGCA-3′ (R) for *β-actin*; 5′ GATGAAATCCACCAAAGCTCAC-3′ (F) and 5′-GCAGTACGGGTCCTCTTGT-3′ (R) for *Pdx1*; 5′-TCACCTTCCCTATCTCTCTGTCTC-3′ (F) and 5′CAATGCTCACTCCTTTCTTAACC-3′ (R) for *Tmem132d*, 5′-TTGATTGGCGTTTGAGATTGG-3′ (F) and 5′-GCTTCAGTATCATGCCATTCC-3′ (R) for *Rpl13*, 5′-AAGTGAGGTGGACATGTTCG-3′ (F) and 5′-GATGCTTCTGTGTGTACGTTG-3′ (R) for *Get4* and 5′-AGTTGCTCGTTCAGTACC-3′ (F) and 5′-GCTTCCTTAGTCAATGTTTTCCAG-3′ (R) for *Ndufa1* gene. Thermal cycling was instigated for 4 min at 94 °C followed by 35 cycles of PCR (94 °C for 15 s; 60 °C for 30 s and 72 °C for 30 s). The mRNA abundance for the genes of interest was quantified using ∆CT method and relatively expressed to β-actin mRNA. Data were expressed as means ± standard error of means. Statistical comparisons were made using Student's *t *test at p value < 0.05.

### Ethics approval

The animal study was reviewed, approved and performed in accordance with relevant guidelines and regulations by Institutional Animal Use and Care Committee of the University of Alabama at Birmingham (IACUC; Animal Project Numbers:10088 and 20653). The study was also carried out in compliance with the Animal Research Reporting of In Vivo experiments (ARRIVE) guidelines.

## Supplementary Information

Supplementary Information 1.

Supplementary Information 2.

Supplementary Information 3.

## References

[1] Seyfried TN, Huysentruyt LC (2013). On the origin of cancer metastasis. Crit. Rev. Oncog..

[2] Siegel RL, Miller KD, Jemal A (2020). Cancer statistics, 2020. CA Cancer J. Clin..

[3] Fu X, Osborne CK, Schiff R (2013). Biology and therapeutic potential of PI3K signaling in ER+/HER2-negative breast cancer. Breast.

[4] Ali S, Coombes RC (2002). Endocrine-responsive breast cancer and strategies for combating resistance. Nat. Rev. Cancer.

[5] Miller A (1978). A study of diet and breast cancer. Am. J. Epidemiol..

[6] Baghurst PA, Rohan TE (1994). High-fiber diets and reduced risk of breast cancer. Int. J. Cancer.

[7] Simopoulos AP (2001). The Mediterranean diets: What is so special about the diet of Greece? The scientific evidence. J. Nutr..

[8] Ramos S (2008). Cancer chemoprevention and chemotherapy: Dietary polyphenols and signalling pathways. Mol. Nutr. Food Res..

[9] Wu AH, Yu MC, Tseng CC, Hankin J, Pike MC (2003). Green tea and risk of breast cancer in Asian Americans. Int. J. Cancer.

[10] Li Y, Buckhaults P, Cui X, Tollefsbol TO (2016). Combinatorial epigenetic mechanisms and efficacy of early breast cancer inhibition by nutritive botanicals. Epigenomics.

[11] Li S, Chen M, Wu H, Li Y, Tollefsbol TO (2020). Maternal epigenetic regulation contributes to prevention of estrogen receptor–negative mammary cancer with broccoli sprout consumption. Cancer Prev. Res..

[12] Raimondi C, Falasca M (2011). Targeting PDK1 in cancer. Curr. Med. Chem..

[13] Zhong S, Johnson DL (2009). The JNKs differentially regulate RNA polymerase III transcription by coordinately modulating the expression of all TFIIIB subunits. Proc. Natl. Acad. Sci..

[14] Fonseca-Sanchéz MA (2013). microRNA-18b is upregulated in breast cancer and modulates genes involved in cell migration. Oncol. Rep..

[15] Iwakawa R (2015). Expression and clinical significance of genes frequently mutated in small cell lung cancers defined by whole exome/RNA sequencing. Carcinogenesis.

[16] Koike, K. *et al.* (AACR, 2019).

[17] Sun Y (2012). Treatment-induced damage to the tumor microenvironment promotes prostate cancer therapy resistance through WNT16B. Nat. Med..

[18] Vitale SG (2016). Peroxisome proliferator-activated receptor modulation during metabolic diseases and cancers: Master and minions. PPAR Res..

[19] Bonecchi R, Mollica Poeta V, Capucetti A, Massara M (2019). Chemokines and chemokine receptors: New targets for cancer immunotherapy. Front. Immunol..

[20] Lima ZS (2019). Recent advances of therapeutic targets based on the molecular signature in breast cancer: Genetic mutations and implications for current treatment paradigms. J. Hematol. Oncol..

[21] Byrareddy SN (2016). Sustained virologic control in SIV+ macaques after antiretroviral and α4β7 antibody therapy. Science.

[22] Solomon KA, Covington MB, DeCicco CP, Newton RC (1997). The fate of pro-TNFalpha following inhibition of metalloprotease-dependent processing to soluble TNF-alpha in human monocytes. J. Immunol..

[23] Chu W-F (2009). Sulforaphane induces G2–M arrest and apoptosis in high metastasis cell line of salivary gland adenoid cystic carcinoma. Oral Oncol..

[24] Bryant CS (2010). Sulforaphane induces cell cycle arrest by protecting RB-E2F-1 complex in epithelial ovarian cancer cells. Mol. Cancer.

[25] Lee Y-R (2019). Reactivation of PTEN tumor suppressor for cancer treatment through inhibition of a MYC-WWP1 inhibitory pathway. Science.

[26] Li Y, Zhang T (2013). Targeting cancer stem cells with sulforaphane, a dietary component from broccoli and broccoli sprouts. Future Oncol..

[27] Li Y (2010). Sulforaphane, a dietary component of broccoli/broccoli sprouts, inhibits breast cancer stem cells. Clin. Cancer Res..

[28] Deb G, Thakur VS, Limaye AM, Gupta S (2015). Epigenetic induction of tissue inhibitor of matrix metalloproteinase-3 by green tea polyphenols in breast cancer cells. Mol. Carcinog..

[29] Thangapazham RL (2007). Green tea polyphenols and its constituent epigallocatechin gallate inhibits proliferation of human breast cancer cells in vitro and in vivo. Cancer Lett..

[30] Wescott MP (2009). Pancreatic ductal morphogenesis and the Pdx1 homeodomain transcription factor. Mol. Biol. Cell.

[31] Galmiche L (2008). Transcription factors involved in pancreas development are expressed in paediatric solid pseudopapillary tumours. Histopathology.

[32] Ma J (2008). Pancreatic duodenal homeobox-1 (PDX1) functions as a tumor suppressor in gastric cancer. Carcinogenesis.

[33] Sakai H (2004). PDX1 homeobox protein expression in pseudopyloric glands and gastric carcinomas. Gut.

[34] Karapetsas A (2015). Overexpression of GPC6 and TMEM132D in early stage ovarian cancer correlates with CD8. BioMed Res. Int..

[35] Shann Y-J (2008). Genome-wide mapping and characterization of hypomethylated sites in human tissues and breast cancer cell lines. Genome Res..

[36] Wallace DC (1999). Mitochondrial diseases in man and mouse. Science.

[37] Nicolaou K (2000). Combinatorial synthesis of novel and potent inhibitors of NADH: Ubiquinone oxidoreductase. Chem. Biol..

[38] Smeitink J, Sengers R, Trijbels F, van den Heuvel L (2001). Human NADH: Ubiquinone oxidoreductase. J. Bioenerg. Biomembr..

[39] Yadava N, Houchens T, Potluri P, Scheffler IE (2004). Development and characterization of a conditional mitochondrial complex I assembly system. J. Biol. Chem..

[40] Raha S, Myint AT, Johnstone L, Robinson BH (2002). Control of oxygen free radical formation from mitochondrial complex I: Roles for protein kinase A and pyruvate dehydrogenase kinase. Free Radical Biol. Med..

[41] Au HC, Seo BB, Matsuno-Yagi A, Yagi T, Scheffler IE (1999). The NDUFA1 gene product (MWFE protein) is essential for activity of complex I in mammalian mitochondria. Proc. Natl. Acad. Sci..

[42] Kobayashi T (2006). Activation of the ribosomal protein L13 gene in human gastrointestinal cancer. Int. J. Mol. Med..

[43] Jung Y (2011). Clinical validation of colorectal cancer biomarkers identified from bioinformatics analysis of public expression data. Clin. Cancer Res..

[44] Akman HB, Oyken M, Tuncer T, Can T, Erson-Bensan AE (2015). 3′ UTR shortening and EGF signaling: Implications for breast cancer. Hum. Mol. Genet..

[45] Dolezal JM, Dash AP, Prochownik EV (2018). Diagnostic and prognostic implications of ribosomal protein transcript expression patterns in human cancers. BMC Cancer.

[46] Guy CT (1992). Expression of the neu protooncogene in the mammary epithelium of transgenic mice induces metastatic disease. Proc. Natl. Acad. Sci..

[47] Königshoff M, Wilhelm J, Bohle RM, Pingoud A, Hahn M (2003). HER-2/neu gene copy number quantified by real-time PCR: Comparison of gene amplification, heterozygosity, and immunohistochemical status in breast cancer tissue. Clin. Chem..

[48] Li Y, Buckhaults P, Li S, Tollefsbol T (2018). Temporal efficacy of a sulforaphane-based broccoli sprout diet in prevention of breast cancer through modulation of epigenetic mechanisms. Cancer Prev. Res..

[49] Bray NL, Pimentel H, Melsted P, Pachter L (2016). Near-optimal probabilistic RNA-seq quantification. Nat. Biotechnol..

[50] Love MI, Soneson C, Robinson MD (2017). Importing transcript abundance datasets with tximport. Dim Txi. Inf. Rep. Sample 1.

[51] Akalin A (2012). methylKit: A comprehensive R package for the analysis of genome-wide DNA methylation profiles. Genome Biol..

[52] Krueger F, Andrews SR (2011). Bismark: A flexible aligner and methylation caller for Bisulfite-Seq applications. Bioinformatics.

[53] Ritchie ME (2015). limma powers differential expression analyses for RNA-sequencing and microarray studies. Nucleic Acids Res..

[54] Wickham, H. & Wickham, M. H. (2007).

[55] Sherman BT (2007). The DAVID Gene Functional Classification Tool: A novel biological module-centric algorithm to functionally analyze large gene lists. Genome Biol..

[56] Liao Y, Wang J, Jaehnig EJ, Shi Z, Zhang B (2019). WebGestalt 2019: Gene set analysis toolkit with revamped UIs and APIs. Nucleic Acids Res..

[57] Li Y, Chen H, Hardy TM, Tollefsbol TO (2013). Epigenetic regulation of multiple tumorrelated genes leads to suppression of breast tumorigenesis by dietary genistein. PLoS One.

[58] Li Y, Yuan Y-Y, Meeran SM, Tollefsbol TO (2010). Synergistic epigenetic reactivation of estrogen receptor-α (ERα) by combined green tea polyphenol and histone deacetylase inhibitor in ERα-negative breast cancer cells. Mol. Cancer.

