# Supplementary material for: Systematic integrated analyses of methylomic and transcriptomic impacts of early combined botanicals on estrogen receptor-negative mammary cancer
Source: Sci Rep. 2021 May 4;11:9481. doi: 10.1038/s41598-021-89131-5 (PMC8096837; doi:10.1038/s41598-021-89131-5)
Supplement: Supplementary file 3 — Supplementary Information 3. [file 41598_2021_89131_MOESM3_ESM.docx]

**Systematic integrated analyses of methylomic and transcriptomic impacts of early combined botanicals on estrogen receptor-negative mammary cancer**

Itika Arora^1^, Yuanyuan Li^2,3^, Manvi Sharma^1^, Michael R. Crowley^4^, David K. Crossman^4^, Shizhao Li^1*^, Trygve O. Tollefsbol^1,5,6,7,8*^

^1^Department of Biology, University of Alabama at Birmingham, 1300 University Boulevard, Birmingham, AL 35294, USA

^2^Department of Obstetrics & Gynecology and Women’s Health, University of Missouri, Columbia, MO 65201, USA

^3^Department of Surgery, University of Missouri, Columbia, MO 65212, USA

^4^Department of Genetics, University of Alabama at Birmingham, Birmingham, AL 35294, USA

^5^Comprehensive Center for Healthy Aging, University of Alabama Birmingham, 1530 3rd Avenue South, Birmingham, AL 35294, USA

^6^Comprehensive Cancer Center, University of Alabama at Birmingham, Birmingham, AL 35233, USA

^7^Nutrition Obesity Research Center, University of Alabama at Birmingham, Birmingham, AL 35294, USA

^8^Comprehensive Diabetes Center, University of Alabama at Birmingham, Birmingham, AL 35294, USA

**Supplementary Figure S1**


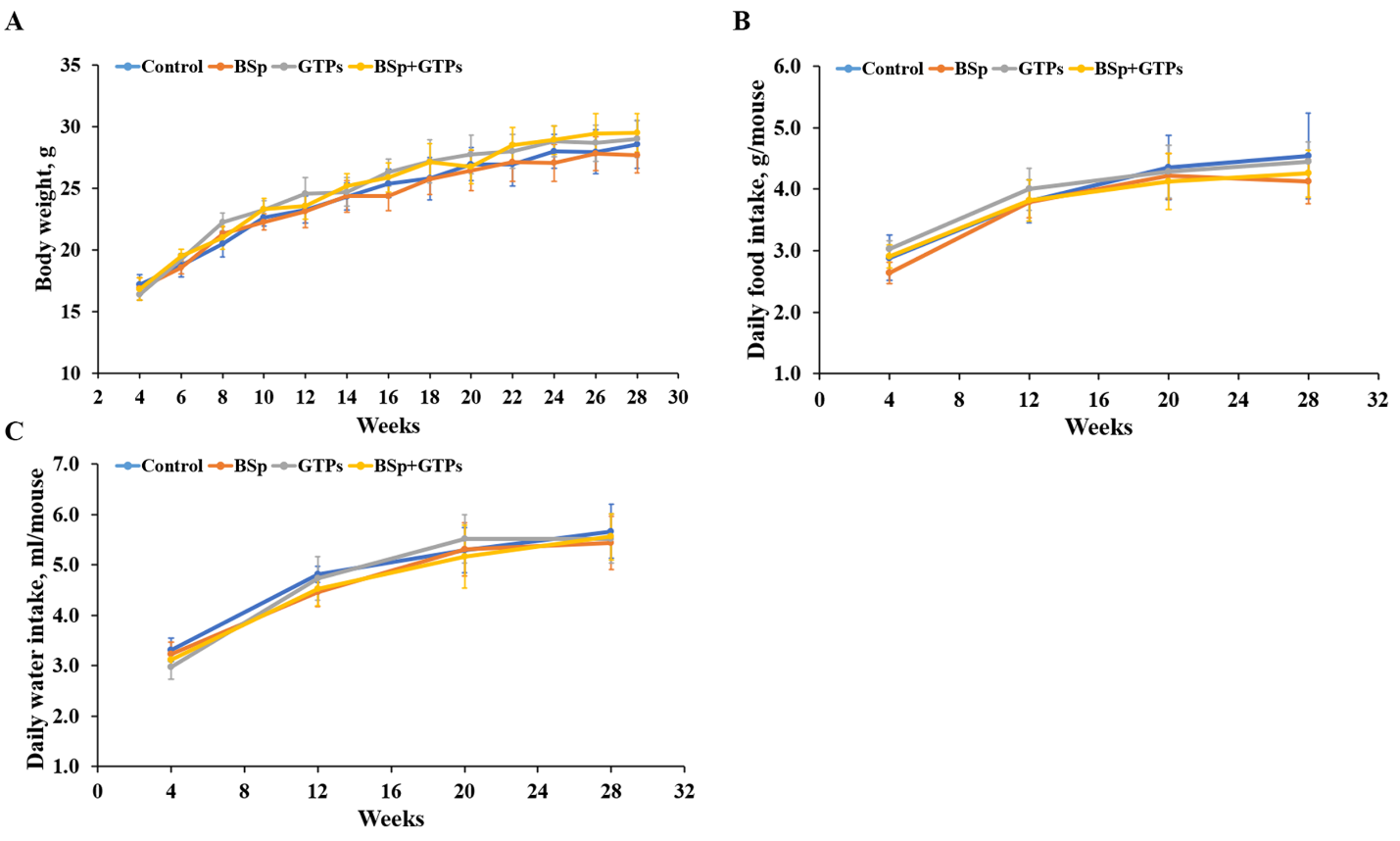


**Supplementary Figure S1. (a)** Mouse body weight food intake. **(b)** Mouse body weight water intake **(c)** Mouse body weight of control female HER2/neu mice and those exposed to BSp, GTPs, or both from weaning through 31 wk of age. Mouse body weight was recorded biweekly from 4 to 28 wk of age. Mouse food and water intakes were recorded at 4, 12, 20 and 28 wk of age. Values are means ± SEMs, n = 15-20. Labeled means, or means at a time, without a common letter differ, *P* < 0.05. BSp, broccoli sprouts; Control, AIN93G diet; GTPs, green tea polyphenols.

**Supplementary Figure S2**


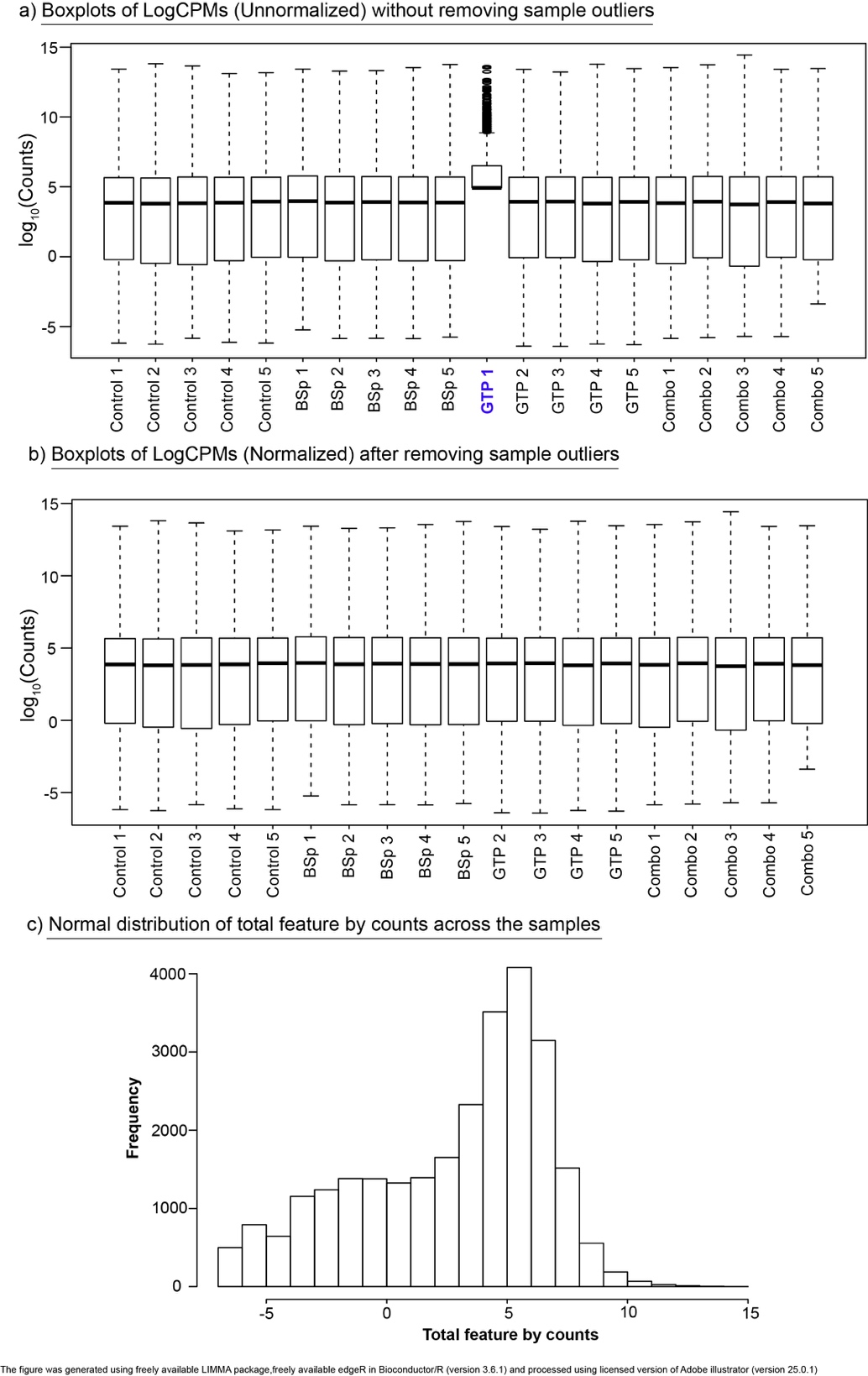


**Supplementary Figure S2. (a)** Boxplot illustrating sample counts across different treatment groups (N_Control_ = 5, N_Bsp_ = 5, N_GTPs_ = 5 and N_Combination_ = 5) before normalization (before removing outlying samples). **(b)** Boxplot illustrating sample counts across different treatment groups (N_Control_ = 5, N_Bsp_ = 5, N_GTPs_ = 4 and N_Combination_ = 5). **(c)** Histogram demonstrating normal distribution of total feature by counts across different samples wherein x-axis represents the total frequency across 20 samples and x-axis represents sample counts.

**Supplementary Figure S3**

**
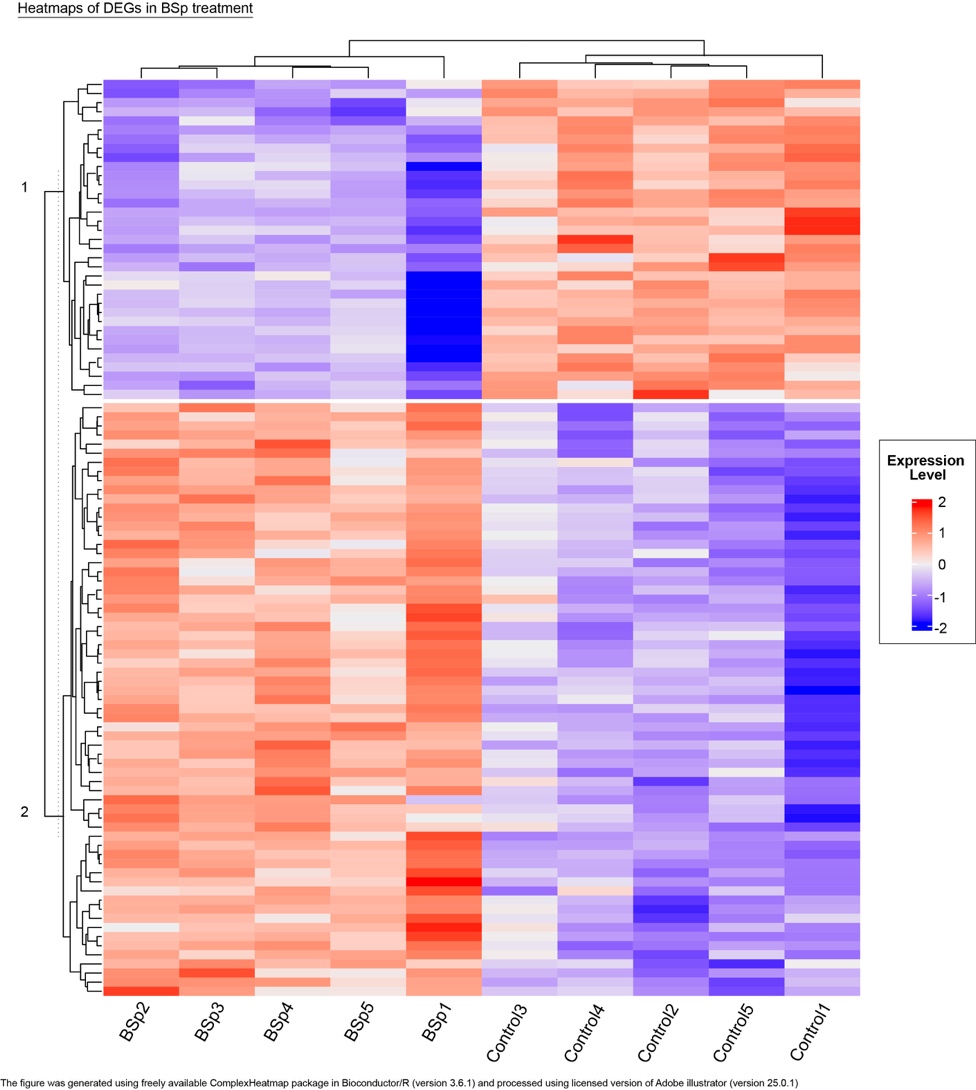
**

**Supplementary Figure S3.** Heatmap representing 146 up-regulated and down-regulated genes in the BSp treatment group based on q-value. Each row corresponds to differentially expressed transcripts and each column represents biological replicates in control (N=5) and BSp (N=5) treatment. Blue color denotes lower expression levels and red color denotes higher expression levels.

**Supplementary Figure S4**

**
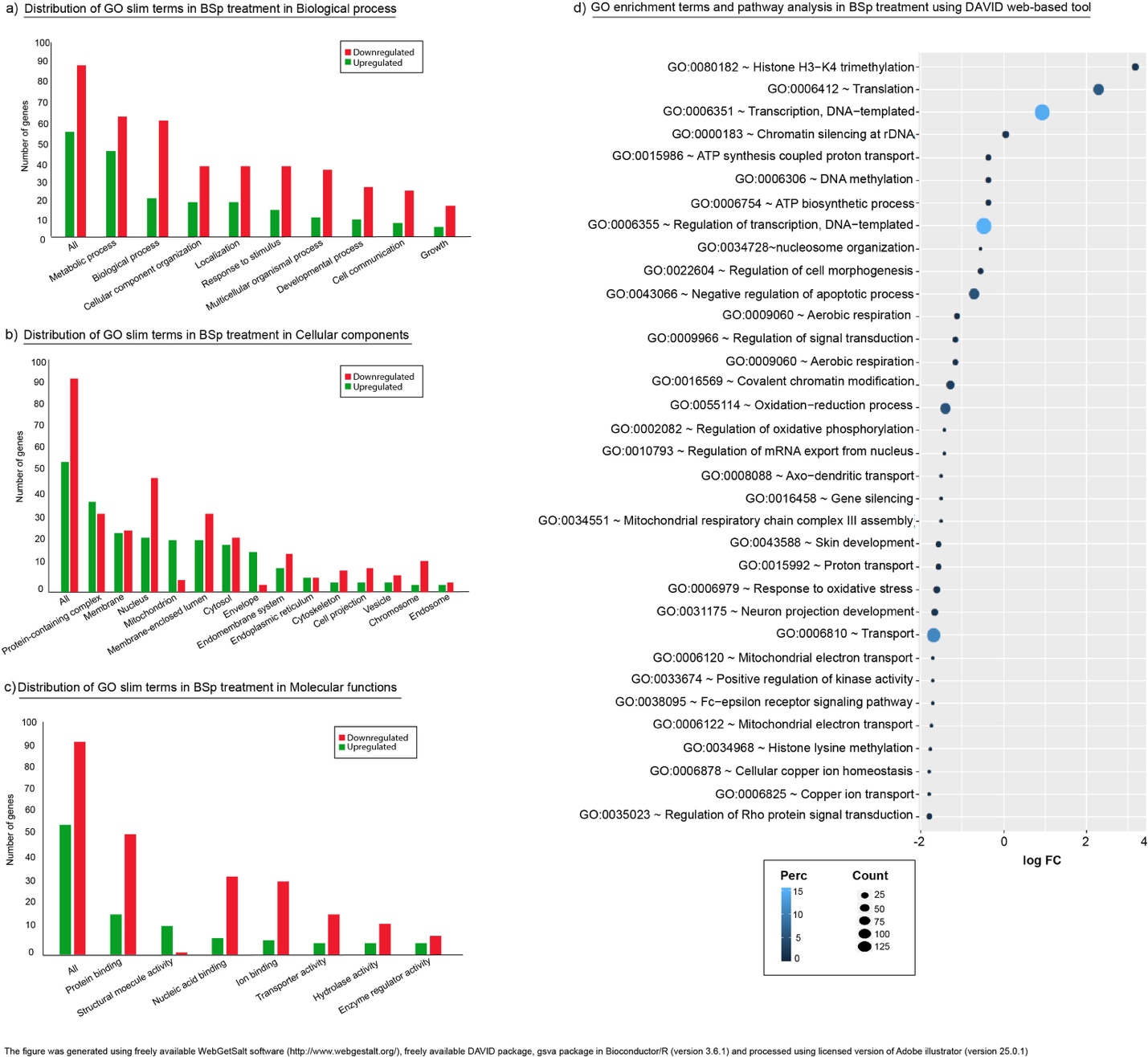
**

**Supplementary Figure S4.** Bar plot distribution of GO slim terms of differentially expressed transcripts related to BSp treatment group in **(a)** biological process, **(b)** cellular components and **(c)** molecular functions wherein red bars represents down-regulated genes and green bars represents up-regulated genes. The height in the bar plot represents the total number of differentially expressed genes. **(d)** Gene ontology enrichment terms and REACTOME pathways analyses using DAVID web-based tool. The plot is sorted based on decreasing Fold change (FC) wherein Y-axis represents specific GO terms related to biological pathways and X-axis represents log_10_(FC) associated with each GO term.

**Supplementary Figure S5**

**
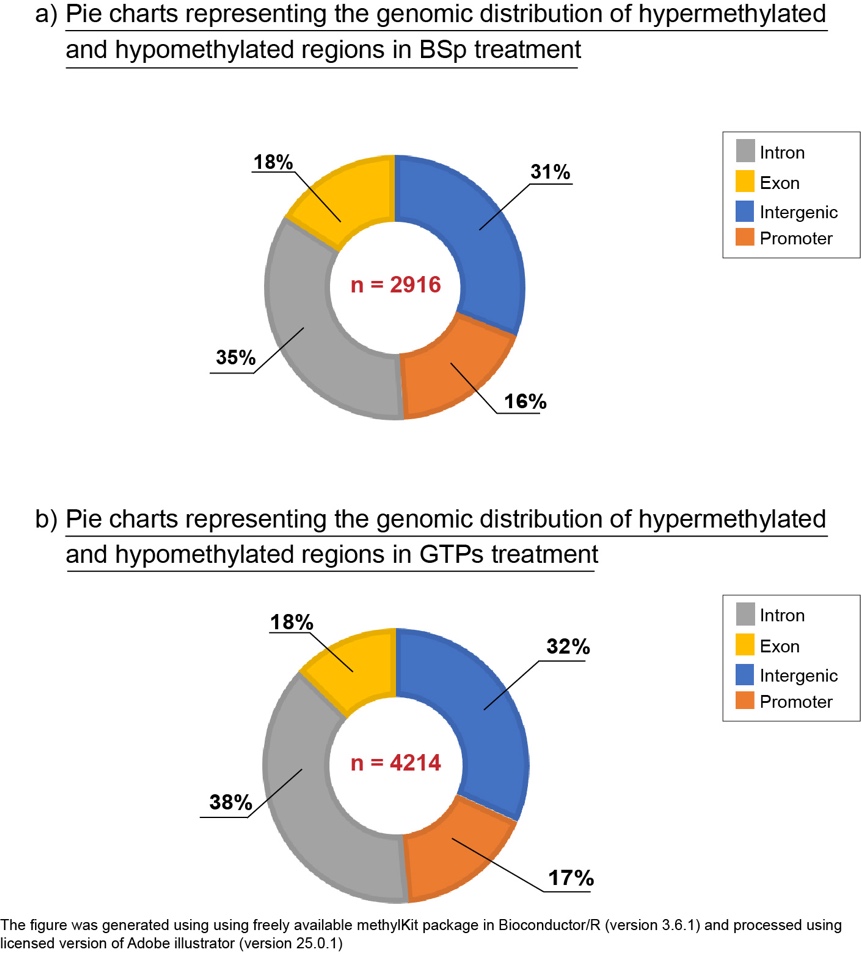
**

**Supplementary Figure S5.** Pie charts representing the genomic distribution of hypo- and hypermethylated regions in **(a)** BSp treatment group (N_BSp_ = 2916) and **(b)** GTPs (N_GTPs_ = 4214) treatment group. Grey color represents intronic regions, yellow color represents exonic regions, blue color represents intergenic regions and orange color represents promoter regions.

**Supplementary Figure S6**

**
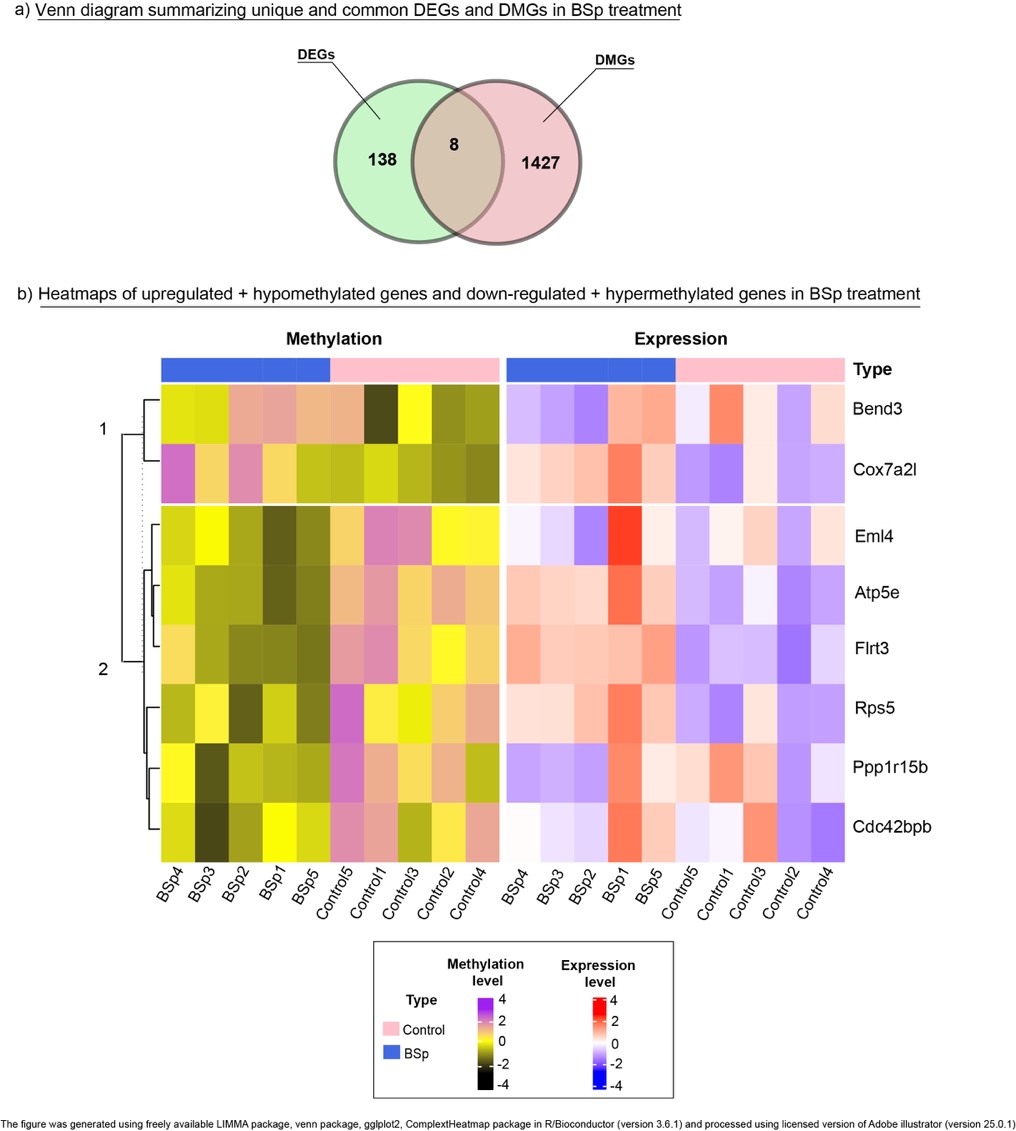
**

**Supplementary Figure S6.** Correlation of DEGs and DMGs in BSp treatment group. **(a)** Venn diagram representing unique and overlapping differentially expressed genes (DEGs) and differentially methylated genes (DMGs) in BSp treatment group. **(b)** Heatmap representing overlapping 8 up-regulated and down-regulated genes in the combination (BSp + GTPs) treatment group based on q-value. Each row corresponds to differentially expressed and differentially methylated transcripts and each column represents biological replicates in control (N=5) and the combination (N=5) treatment group. Blue color denotes lower expression levels and red color denotes higher expression levels.

**Supplementary Table S1.** Reference list of transcripts with P-value and fold change in BSp treatment group**.**

**Supplementary Table S2.** A list of differentially expressed (DE) genes in BSp treatment (p<0.05) and calculated fold change at transcriptome level.

**Supplementary Table S3**. Reference list of transcripts with P-value and fold change in GTPs treatment group.

**Supplementary Table S4**. Reference list of transcripts with P-value and fold change in GTPs treatment group.

**Supplementary Table S5**. A list of differentially expressed (DE) genes in the combination (BSp + GTPs) treatment group (P-value<0.05) and calculated fold change at transcriptome level.

**Supplementary Table S6.** Reference list of methylation level changes in BSp treatment group.

**Supplementary Table S7**. Reference list of transcripts that are differentially expressed genes (P-value<0.05) and differentially methylated (methylation difference) in BSp treatment group.

**Supplementary Table S8**. Unique differentially expressed genes (P-value<0.05) and differentially methylated (methylation difference) in BSp treatment group.

**Supplementary Table S9**. Reference list of methylation level changes in GTPs treatment group.

**Supplementary Table S10**. Reference list of methylation level changes in the combination (BSp + GTPs) treatment group.

**Supplementary Table S11**. Reference list of transcripts that are differentially expressed genes (p<0.05) and differentially methylated (methylation difference) in the combination (BSp + GTPs) treatment group.

**Supplementary Table S12.** Unique differentially expressed genes (P-value<0.05) and differentially methylated (methylation difference) in the combination (BSp + GTPs) treatment group.

**List of abbreviations**

BSp = Broccoli sprouts

BC = Breast cancer

Combo = Combination

GTPs = Green tea polyphenols

*Get4* = Guided Entry of Tail-Anchored Protein Factor 4

DAVID = Database for Annotation, Visualization and Integrated Discovery

DMGs = Differentially methylated genes

DEGs = Differentially expressed genes

DMRs = Differentially methylated regions

DNA = Deoxyribonucleic acid

EGCG = Epigallocatechin gallate

ER(-) BC = Estrogen receptor negative breast cancer

FC = Fold change

FDR = False discovery rate

IACUC = Institutional Animal Use and Care Committee of the University of Alabama at Birmingham

*JNK1* = C-jun N-terminal kinases

KEGG = Kyoto Encyclopedia of Genes and Genomes

mm = Mus musculus

*Ndufa1* = NADH: Ubiquinone Oxidoreductase Subunit A1

qRT-PCR = Quantitative reverse transcription PCR

QC = Quality control

*Tmem132d* = Transmembrane protein 123D

TNBC = Triple negative breast cancer

PCoA = Principal component analyses

*Pdx1* = Pancreatic duodenum homeobox protein 1

PR = Progesterone receptor

*Rpl13* = Ribosomal Protein L13

RNA = Ribonucleic acid

RNA-seq = RNA sequencing

RRBS = Reduced representation bisulfite sequencing

WebGestalt = WEB-based GEne SeT Analyses
